# Volatile pyrethroid spatial repellents for preventing mosquito bites: a systematic review and meta-analysis

**DOI:** 10.1016/j.ebiom.2025.105891

**Published:** 2025-08-26

**Authors:** Ingrid Chen, Sarah L. Miller, Daniel Msellemu, Aidi G. Lugenge, Johnson Kyeba Swai, Nicole Achee, Marta Andrés, Christopher S. Bibbs, Theeraphap Chareonviriyaphap, J. Derek Charlwood, Greg Devine, Noel Elman, Ulrike Fillinger, Carmen Flores-Mendoza, Seth Gibson, Nicodem Govella, Steven Gowelo, Sebastian Horstmann, Hitoshi Kawada, Daniel Kline, Aaron Lloyd, Neil F. Lobo, Marta F. Maia, Arnold Mmbando, Mara Moreno-Gómez, Amy C. Morrison, Winifrida Mponzi, Emmanuel P. Mwanga, Margaret Njoroge, Sheila B. Ogoma, Fredros O. Okumu, Mercy Opiyo, Welbeck A. Oumbouke, John Paliga, Arissara Pongsiri, Alongkot Ponlawat, Manop Saeaung, Ferdinand Salazar, Onyango Sangoro, Jennifer C. Stevenson, Chutipong Sukkanon, Din Syafruddin, Mgeni Mohamed Tambwe, Julie-Anne A. Tangena, Elodie A. Vajda, Gonzalo Vazquez-Prokopec, Joseph M. Wagman, Chanly Yan, Isabel Elaine Allen, Sarah J. Moore

**Affiliations:** a Department of Epidemiology and Biostatistics, University of California, San Francisco, CA, USA; b Malaria Elimination Initiative, Institute for Global Health Sciences, University of California, San Francisco, CA, USA; c Ifakara Health Institute, Bagamoyo, Tanzania; d Department of Biological Sciences, Eck Institute for Global Health, University of Notre Dame, Notre Dame, IN, USA; e University College London, London, UK; f National Center National Institute for Agricultural and Food Research and Technology, Spanish National Research Council, Valdeolmos, Spain; g Salt Lake City Mosquito Abatement District, Salt Lake City, UT, USA; h Department of Entomology, Faculty of Agriculture, Kasetsart University, Bangkok, Thailand; i Global Health and Tropical Medicine, Instituto de Higiene e Medicina Tropical, Universidade Novo de Lisboa, Lisbon, Portugal; j QIMR Berghofer Medical Research Institute, Herston, Australia; k GearJump Technologies LLC, Boston, MA, USA; l U.S. Naval Medical Research Unit SOUTH, Lima, Peru; m U.S. Department of Agriculture, Agricultural Research Service, Center for Medical, Agricultural, and Veterinary Entomology, Gainesville, FL, USA; n Envu, 2022 ES Deutschland GmbH, Monheim am Rhein, Germany; o Institute of Tropical Medicine, Nagasaki University, Nagasaki, Japan; p Lee County Mosquito Control District, Buckingham, FL, USA; q Nuffield Department of Medicine, Centre for Tropical Medicine and Global Health, University of Oxford, Oxford, UK; r Department of Biosciences, KEMRI Wellcome Trust Research Programme, Kilifi, Kenya; s Ifakara Health Institute, Ifakara, Morogoro, Tanzania; t Department of Biosciences, Durham University, Durham, UK; u Henkel, Barcelona, Spain; v Department of Pathology, Microbiology, and Immunology, School of Veterinary Medicine, University of California, Davis, CA, USA; w International Centre for Insect Physiology and Ecology (icipe), Nairobi, Kenya; x PMI VectorLink, Abt Associates, Nairobi, Kenya; y University of Glasgow, Glasgow, Scotland, UK; z Department of Vector Biology, Liverpool School of Tropical Medicine, Liverpool, UK; aa Department of Medical Entomology, Research Institute for Tropical Medicine, Department of Health, Alabang, Muntinlupa City, Philippines; ab Department of Clinical Microscopy, Faculty of Medical Technology, Mahidol University, Salaya, Phuttamonthon, Nakhon Pathom, Thailand; ac Department Parasitology, Faculty of Medicine, Hasanuddin University, Makassar, Indonesia; ad Swiss Tropical and Public Health Institute, Allschwil, Switzerland; ae Department of Environmental Sciences, Emory University, Atlanta, GA, USA; af Independent Consultant, Disease Ecology and Vector Control, Mannheim, Germany; ag Wagman Global Health Consulting, Rockville, MD, USA

**Keywords:** Meta-analysis, Vector control, Spatial repellent, Spatial emanator, Mosquito, Volatile pyrethroid

## Abstract

**Background:**

Volatile pyrethroid spatial repellents (VPSRs) can prevent mosquito-borne diseases including malaria and dengue fever, but the use of varied evaluation methods has resulted in a lack of clarity regarding their protective efficacy (PE) against contact with mosquitoes. This systematic review and meta-analysis consolidates the entomological evidence base on the PE of VPSRs against *Anopheles*, *Aedes*, and *Culex* mosquitoes and different test methods used.

**Methods:**

We identified studies completed between January 2000 and September 2023 by searching through databases, conference abstracts, and personal correspondences. Included studies were semi-field or field studies that measured the PE of VPSRs using human landing catch (HLC) of mosquito landings on human legs and/or mosquito trap density, the number of mosquitoes captured using traps per set time period, compared to control groups. The systematic review summarised study-level data using a generalised linear mixed model with random effects. The meta-analysis pooled individual mosquito-level data and weather data on temperature, humidity, and wind from satellites, analysing PE subgrouped by product format, active ingredient, mosquito capture method used, mosquito species, and indoor vs outdoor setting. Risk of bias was assessed using a SYRCLE tool adapted for mosquito studies. Additional studies published from October 2023 to July 2025 were summarised. PROSPERO registration: CRD42021268852.

**Findings:**

58 eligible publications showed that VPSRs provided an average of 56% (95% CI 50, 62%) PE from mosquito bites. Meta-analysis of individual mosquito-level data from 50 (86%) of eligible studies involving 1,703,120 mosquitoes showed that PE was highest when measured using HLC, with similar results seen in semi-field (58%, 95% CI 54, 62%) and field studies (50%, 95% CI 40, 59%). Differences between indoor (54%, 95% CI 18, 68%) and outdoor settings (56%, 95% CI 51, 60%) were unclear. Species-level differences were observed with low PE seen in *Anopheles funestus* (31%, 95% CI 19, 43%); the potential for cross-resistance to solid-state pyrethroids is unclear. Efficacy was not sensitive to combined weather effects.

**Interpretation:**

VPSRs offer protection from contact with mosquitoes, with semi-field studies reflecting field data and species-level differences observed. HLC provided the best quality data. Additional field studies that evaluate outdoor protection in malaria-endemic settings are needed, especially in West African, South American, and Southeast Asian settings.

**Funding:**

10.13039/100000002National Institutes of Health (10.13039/100000060National Institute of Allergy and Infectious Diseases (K01AI156182)) and “Accelerate to Eliminate Malaria” program.

**Research in context**

**Evidence before this study**

We systematically searched PubMed articles published through July 28, 2025, for systematic reviews and meta-analysis studies on the entomological efficacy of volatile pyrethroid spatial repellents (VPSRs) and consulted with experts seeking reviews that were not found on PubMed. We included review studies on the entomological efficacy of VPSRs of any format on *Anopheles*, *Aedes*, and/or *Culex* mosquitoes. Using search terms “spatial repellent review” we identified 49 full-text articles in PubMed, three of which met our inclusion criteria: a 2012 systematic review on mosquito coils and passive emanators, a 2017 review on pyrethroid-containing spatial repellents that investigated mosquito mortality, knockdown, blood-feeding inhibition, and deterrence, and a 2021 scoping review on insecticide-treated window screens and eaves that examined PE, mosquito mortality, and deterrence. A 2020 expert review on spatial repellents that was not peer reviewed investigated those same outcomes in addition to PE and mosquito oviposition. None of these reviews synthesised original mosquito data from studies, and heterogeneity across studies reflecting various VPSR formats, active ingredients, study types, use cases (indoor vs outdoor), mosquito species, and entomological endpoints prevented the generation of quantitative PE estimates on VPSRs, and which factors influence their efficacy.

**Added value of this study**

This systematic consolidation of the evidence base demonstrates the efficacy of VPSRs across multiple settings and mosquito species, including data at an individual mosquito level as well as weather data from study stations and satellites. We provide quantitative data comprising 86% of the published evidence base to clarify the PE of VPSRs and which factors influence their efficacy. Our findings can inform evaluation methods, for which guidelines will be updated following the World Health Organisation recommendation on spatial emanators made on August 13 2025, and can also inform implementation of these products and identify future areas of research needed.

**Implications of all the available evidence**

This meta-analysis shows that VPSRs offer protection from mosquito contact, being efficacious against all *Anopheles*, *Aedes*, and *Culex* mosquito species tested and therefore offering protection from the mosquito vectors of malaria, dengue and Zika, and West Nile virus, respectively. This confirms that VPSRs can be used when there are gaps in protection from mosquito bites, including when individuals at risk spend time outdoors, in peri-domestic settings, are indoors when not using insecticide-treated nets, or are in humanitarian emergencies. Rollout should consider vector species diversity to reflect methods for dengue globally, and for malaria be segmented to 1) East and Southern Africa; 2) West Africa; 3) Southeast Asia; 4) Central and South America; and 5) South Asia, the Middle East, and North Africa. For testing methodologies, the development of standardised methodology will help to reduce the heterogeneity of future evidence generation and should use direct measures of disease exposure such as HLC if possible, with semi-field studies offering more controlled assessments with disease-free mosquitoes, providing a safe means of evaluation. More field studies investigating VPSR use outdoors are needed, especially for forthcoming commercial products, and further investigations are required to establish low-cost methods for monitoring VPSR efficacy over time in operational settings. For geography, more field data are needed from West Africa, South America, and Southeast Asia, where the burden of mosquito-borne disease is high but studies on VPSRs are limited.

## Introduction

More than 700,000 deaths annually are attributed to vector-borne diseases.^1^ The highest burden is from malaria transmitted by female *Anopheles* mosquitoes with an estimated 263 million cases and 597,000 deaths in 2023,^2^ followed by dengue spread by *Aedes* mosquitoes, with over 14 million infections and 10,000 deaths reported in 2024.^3^ Intensified malaria vector control at the beginning of the century has resulted in around 2.2 billion malaria cases and 12.7 million malaria deaths averted between 2000 and 2023.^2^ This is mainly attributed to the scale-up of malaria vector control through the mass distribution of insecticide treated nets (ITNs),^4^ indoor residual spraying (IRS) of insecticides, and larval source management (LSM) for dengue.^5^ However, these interventions have operational challenges. ITNs protect people indoors during sleeping hours and global coverage is insufficient,^6^ IRS is logistically challenging to implement with a decreasing number of programs implementing this strategy at scale,^4^ and LSM requires meticulous planning and community engagement to be cost-effective.^7^ WHO emphasises that additional tools are needed to achieve incremental gains addressing the expanding range of malaria vectors, their behaviours, and gaps in protection from ITNs, IRS and LSM for malaria,^3^^,^^4^^,^^8^ and are also needed to prevent arboviruses spread by *Aedes* and *Culex*-species mosquitoes.^9^

There are many promising vector control tools in the pipeline that may overcome existing challenges^10^^,^^11^ including genetically modified mosquitoes, attractive targeted sugar baits, endectocides, and volatile pyrethroid spatial repellents (VPSRs) also referred to as spatial emanators, which are the focus of this review. VPSRs are devices that continuously disperse an volatile pyrethroid active ingredient into the air to create a bubble of protection from mosquito vectors due to their impact on several mosquito behaviours^12^ including host detection, landing, blood-feeding, survival, and reproduction.^13^ VPSRs include mosquito coils and vaporisers that are widely used representing USD 1.8 billion each year in consumer sales,^14^ as well as newer passive emanator formats that continuously release an active ingredient without the use of heat or electricity, providing longer-lasting protection without the need for daily intervention or compliance by the end-user.

The spatial repellent intervention class recently received a WHO policy recommendation^15^ based on clinical evidence on their efficacy against malaria available from trials in China,^16^ Indonesia,^17^ and Kenya.^18^ This WHO recommendation will trigger a review of entomology testing guidelines^19^ challenged by a heterogeneous evidence base, resulting in a lack of clarity around how to measure the PE of VPSRs and how to harmonise testing methodologies. This study seeks to provide clarity around these challenges through consolidating the entomological evidence base on VPSRs, clarifying their efficacy by pooling original mosquito-level data, investigating factors that affect measured efficacy, and providing insight on the relative performance of various testing methods to inform guidelines as this intervention class is now recommended for its effectiveness to prevent mosquito-borne disease in public health efforts.

## Methods

### Search strategy and selection criteria

For the systematic review, we searched three databases and registries: MEDLINE, Embase, and Web of Science, as well as conference proceedings and correspondences with experts to identify entomological VPSR studies completed between January 1, 2000 and September 6, 2023. The Population, Intervention Comparison, Outcome (PICO) framework with inclusion criteria for the study is shown in Table 1. For databases and registers, search terms were “volatile pyrethroid”, “transfluthrin”, “metofluthrin”, “allethrin”, “prallethrin”, “meperflutherin”, “spatial repellent”, “emanator”, “push-pull”, “coil”, “eave ribbon”, “mosquito”, “vector control”, “bite prevention”, “anopheles”, “aedes”, culex”, “malaria”, “dengue”, and “infectious disease”. For other search methods, we looked at citations from included publications and reviews identified through the literature search and sought conference proceeds from the American Society of Tropical Medicine and Hygiene annual meetings from 2018 onwards, the Multilateral Initiative on Malaria (MIM) meeting in 2018, and the Entomological society of America meetings from 2017 onwards. When contacting authors for study-level data for the meta-analysis, we asked if they completed additional studies that may be eligible for inclusion in this meta-analysis.

**Table 1:** Inclusion criteria using population, intervention, comparison, outcome (PICO) framework.

PICO category	Inclusion criteria	Description
Population	Mosquito species	Measurement of *Anopheles*, *Aedes*, or *Culex* mosquitoes.
Intervention	Volatile pyrethroid spatial repellent (VPSR) use	Any delivery format that contains any of the following active ingredients: metofluthrin, allethrin, prallethrin, transfluthrin, and/or meperfluthrin.
	Semi-field or field studies	Semi-field studies evaluate VPSRs in controlled outdoor environments that simulate natural conditions. Using screened enclosures or similar setups, semi-field systems allow assessment of multiple mosquito responses including mosquito landing or feeding, knockdown and mortality under realistic but standardised conditions. Field studies take place in natural environments.
	Study design to control for bias	Design must control for spatial heterogeneity and variability between human participants which are common sources of bias in entomology studies. This requires rotations of intervention vs control in testing locations as well as mosquito collectors if human landing catch (HLC) is used. Latin square and cross-over studies were included, and all other study designs were assessed individually.
Comparison	Protective entomological efficacy of VPSRs comparing intervention to control (no volatile pyrethroid)	Control can be no intervention or placebo.
Outcome	Measure of mosquito landing using human landing catch (HLC) or mosquito trap density	Primary outcome is required for inclusion: these are studies that measured reductions in mosquito contact based on HLC of mosquito landings on human legs (the gold standard) and/or mosquito trap density, the number of mosquitoes captured using traps per set time period. In this study, we consider experimental huts, which are vector control testing tools designed to mimic local houses, to be a trap measuring mosquito entry and exit. Experimental huts can be used in near-natural field settings and semi-field systems to measure the efficacy of indoor applications of VPSRs. Secondary outcomes are additional entomological assessments (defined in Table 2, among these only protective efficacy is required for inclusion).
Additional criteria	Language	Studies published in the English language.

**Table 2:** Definitions of entomological outcomes measured in bioassays of volatile pyrethroid spatial repellents.

Entomological outcome	Definition
Blood feeding inhibition	Reduction in the number of or the proportion of blood-fed mosquitoes caused by sublethal exposure to active ingredients.
Deterrence	Inhibition of entry due to sublethal exposure to active ingredients i.e., mosquitoes not entering a treated house.
Fecundity reduction	Decrease in the number of viable eggs produced by a blood fed adult female mosquito due to sublethal effects of active ingredients.
Knockdown	Mosquitoes that are incapacitated (unable to move, stand or fly in a coordinated manner) after sublethal exposure to active ingredients measured at set timepoints, typically 60 min after exposure.
Landing inhibition	Reduction in landings in the treatment arm relative to the control arm, typically measured by HLC.
Mortality	Proportion of mosquitoes that are dead, immobile, or incapacitated (unable to move, stand or fly in a coordinated manner) after exposure to the active ingredient measured at set timepoints, typically 24 h after collection.
Non-contact irritancy (repellency or non-contact excito-repellency)	The directional or non-directional movement of adult female mosquitoes away from treated spaces due to sublethal non-tarsal contact with active ingredients i.e., mosquitoes exiting a treated house.
Protective efficacy (PE)	Protection elicited by a vector control tool, in this review measured by reduction in landing or reduction in blood-fed mosquitoes in the treatment arm relative to no treatment (negative control).

Study titles identified through searches were screened by one individual who identified abstracts, full-text articles, and protocols to be screened by two individuals, with discrepancies settled by a third co-author. For the systematic review, eligible studies were analysed based on published data. For the meta-analysis, individual mosquito-level data was sought for all studies included in the systematic review by contacting lead authors up to five times using email. For those willing to share original individual mosquito-level data, data sharing agreements were prepared if requested. Included studies were eligible for the systematic review and those that provided individual mosquito-level data were eligible for meta-analysis. Data analysis methods are described below. Additional studies published between September 7, 2023 and July 28, 2025 that were eligible for inclusion were identified, and their study details and findings were summarised in a table. The study was registered (PROSPERO CRD42021268852).

### Data analysis

The systematic review entailed extracting study details, methods used, and resulting efficacy from peer-reviewed publications and shared data if available. For the study-level forest plot, when published PE estimates were available, a one-step analysis was used, otherwise a two-stage approach was applied by generating estimates for individual studies.

The meta-analysis of individual mosquito-level data entailed standardising and consolidating shared datasets on mosquitoes in a master database to generate pooled estimates on PE grouped by intervention format, active ingredient used, mosquito capture method used, indoor vs outdoor setting, mosquito species affected, and study type (semi-field vs field). This was a one-stage analysis with data extracted including the author and year of publication, country, intervention format, active ingredient, capture method, trap type, setting, study type (semi-field vs field), treatment allocation, mosquito counts, mosquito species, experimental day, mosquito collector ID, temperature, humidity, and hut/chamber/location ID.

The primary outcome for the study was the PE, defined as 1—relative rate associated with intervention group from regression analysis. PE was based on HLC, the gold standard for mosquito entomological efficacy studies where a trained human collector sits with exposed lower legs and uses an aspirator to collect mosquitoes that land before they feed with the intent to bite, and/or trap density which refers to the number of mosquito captured per trap over a defined period, usually per trap per night, with trap type noted.

Secondary outcomes included landscaping the measurement of blood feeding inhibition, deterrence, fecundity reduction, knock-down, mortality, and non-contact irritancy (defined in Table 2), as well as mosquito resistance status to solid-state pyrethroids where information was available. For outdoor studies, satellite data on temperature, humidity, and wind on efficacy was extracted from Terraclimate,^20^ a global dataset of monthly climate data with 4-km spatial resolution, for sensitivity analysis. Weather data was considered unapplicable to studies in indoor settings. Sensitivity analysis also investigated the effect of removing one study at a time on pooled efficacy outcomes.

Risk of bias (RoB) was assessed using a modified version of SYRCLE's RoB Tool^21^ designed for animal intervention studies based on Cochrane methods.^21^ The SYRCLE tool contains ten entries related to the following areas of bias: selection, performance, detection, attrition, reporting, and other. Since these criteria were not directly applicable to mosquito studies, we created the modified RoB tool for mosquito studies described below, retaining the domains used in both the Cochrane and SYRCLE tools on selection bias, blinding, reporting bias, and other sources of bias. Our modified RoB tool includes randomisation, blinding, completeness of outcome reporting, mosquito conditions for semi-field studies, and funder involvement, which is explained in the following paragraphs.

Randomisation for spatial and temporal heterogeneity; we required this category for inclusion in our current study but included it in this tool for future assessments of bias in entomology studies involving mosquitoes. For cluster-randomised studies only, we assessed whether the distribution of relevant baseline characteristics were balanced between the intervention and control groups. This was considered low risk of bias if characteristics were similar (e.g., gender, age, use of other interventions, socioeconomic status, housing, occupation) or if despite baseline differences, treatment and control were rotated between locations, and high risk of bias if there were baseline differences, or the treatment and control were not rotated.

Blinding of participants, investigators, and statistician(s); for participant blinding (e.g., HLC volunteers or people collecting mosquitoes from traps), this was considered low risk of bias if participants were blinded to treatment allocation, unclear risk of bias if the control consisted of no intervention and hence was not blinded, and high risk of bias per the judgement of the individual assessing for risk of bias, who noted their concerns to discuss with the study team who then made a joint assessment. For investigator blinding, this was considered low risk of bias if investigators were blinded to treatment allocation, or unclear risk of bias if they were not blinded or if details were not provided in the paper. For statistician blinding, this was considered low risk of bias if statisticians were blinded to treatment allocation, or unclear risk of bias if statisticians were not blinded.

Completeness of outcome reporting; this was considered low risk of bias if all methods on HLC and/or trap density outcomes were reported in the results, or unclear risk of bias if the missing data was due to low numbers. For other cases, this was discussed case by case, noting what was missing and why if data was available. For field studies, for species level composition, risk of bias was considered based on whether the denominator changed by much. If missing data were for other reasons, we described and discussed these, assigning a high risk of bias unless consensus indicated otherwise.

Mosquito conditions for semi-field studies; this was considered low risk of bias if mosquitoes were sugar starved and nulliparous, unclear risk of bias if mosquitoes were not sugar starved and/or not nulliparous, or high risk of bias if mosquitoes were not sugar-starved and nulliparous, or nothing was mentioned regarding this.

Funder involvement; this was considered low risk of bias if authors stated that funders were not involved in study implementation and analysis, high risk of bias if they stated that funders were involved in study implementation and analysis, or unclear risk of bias if funder involvement was not mentioned.

Each study was assessed as having low, unclear, or high risk of bias in each of these categories. The individual assessing for risk of bias highlighted areas where they were unclear on their assessments, after which three to four investigators who assessed for risk of bias convened in a meeting to discuss these unclear areas until consensus on the assessment was reached.

### Ethics

This was a systematic review and meta-analysis using data on mosquitoes; therefore, no ethical approval for human subject research was required.

### Statistics

For both study-level and pooled estimates of the primary outcome, a random effects model using a negative binomial distribution was used, as this model that accounts for overdispersion inherent to mosquito data. The model provided a weighted mean PE with a 95% confidence interval. Experimental day was the random effect, fixed effects were mosquito counts and treatment allocation. Variability between studies was measured using I^2^. Publication bias was assessed using a funnel plot^22^ and Begg's test statistic.^23^

### Role of funders

The funder of the study had no role in study design, data collection, data analysis, data interpretation, or writing of this report.

## Results

**Fig. 1:**
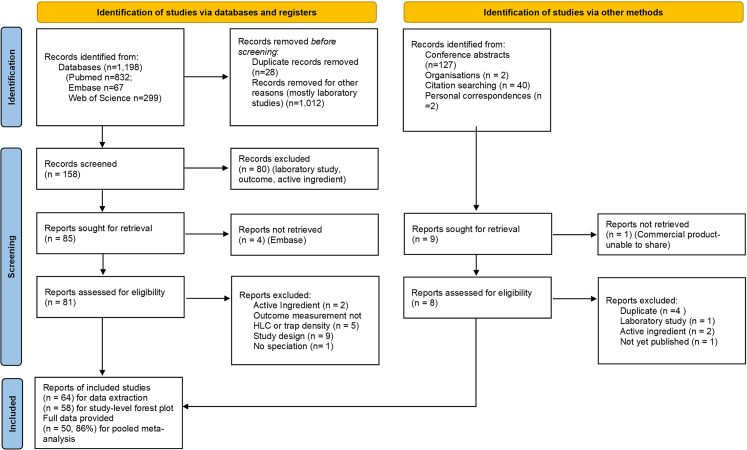
**Flow diagram of studies identified and assessed for inclusion eligibility.** PRISMA chart shows literature search results on the left and grey literature search findings on right, including reasons for exclusion throughout the screening process. Study databases and registers identified 1198 abstracts and 171 studies were identified via other methods. After screening, 64 reports were included for data extraction in tables, 58 were included in the study-level forest plot, and 50 received original mosquito-level data that were pooled in the meta-analysis.

Our search for studies published between January 1, 2000 and September 6, 2023 identified 1369 titles, 1198 from databases (Pubmed, Embase, and Web of Science; specific search strings and dates are detailed in the Appendix Section 1) and 171 from other sources (conference abstracts, consultation with organisations, citation searching, and personal correspondences) (Fig. 1). Of these titles, 158 abstracts were screened, 94 reports were sought for retrieval, and 89 full-text studies or protocols (81 from databases and 8 from other sources) were successfully retrieved and assessed for eligibility. These resulted in 64 studies eligible for inclusion for the systematic review, all of which were published. The main reasons for exclusion were laboratory-based studies, those that used arm-in-cage assays, and those using non-pyrethroid-class active ingredients. Studies that we expected to be included but were not eligible due to before-after study design are listed in Appendix Section 2.

For all 64 included studies, study details were extracted in tables. PE for the forest plot was estimated from 58 of these studies; six studies for which PE could not be estimated are listed in Appendix Section 2. Publications with more than one experiment (e.g., active ingredient or study type) were separated as multiple studies (details in Appendix Section 2) for a total of 84 studies. For the pooled meta-analysis, individual mosquito-level data were from 50 author datasets: 86% of the 58 published studies from the systematic review. Authors from four studies did not reply to email requests for data, and for another four studies published 2013 or earlier, data could not be found (listed in Appendix Section 2).

**Fig. 2:**
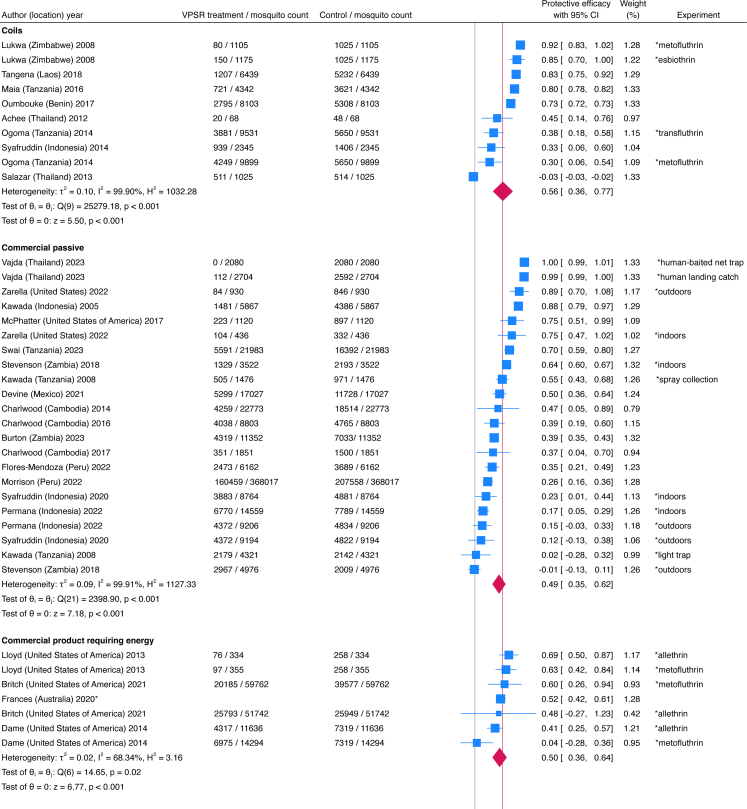
**Study-level forest plotof VPSR protective efficacy subgrouped by intervention format (Part 1).** Each line represents one study. Average protective efficacy signified by red line based on 1,388,993 mosquitoes (84 studies, combining 39 studies in figure 2 and 45 in Fig. 3). Point estimates are blue boxes sized by weight including confidence intervals (horizontal blue line); exact values for these are provided in the column titled ‘Protective efficacy with 95% CI’. Point estimates to the left of the red line show lower efficacy than average, those to the right have higher efficacy. Some publications had more than one experimental study; the column ‘Experiment’ indicates variables specific to that study. Red diamonds show the average efficacy for each product format. The ‘Weight (%)’ column corresponds to the percentage that each study contributes to the average protective efficacy (total 100% when adding values from figures 2 and 3). The test of θi = θj is a test of homogeneity that uses Cochran's Q test (chi-square), values of p < 0.05 indicates that the studies are statistically different from one another. The test of θ = 0 is a z-statistic based on a Wald test that assesses whether overall pooled effects in a group of studies is equal to zero; values of p < 0.05 indicate that overall effects are statistically significantly not equal to zero. These two θ tests are computed for each subgroup. This figure is divided into two parts, with the second half presented in Fig. 3.

**Fig. 3:**
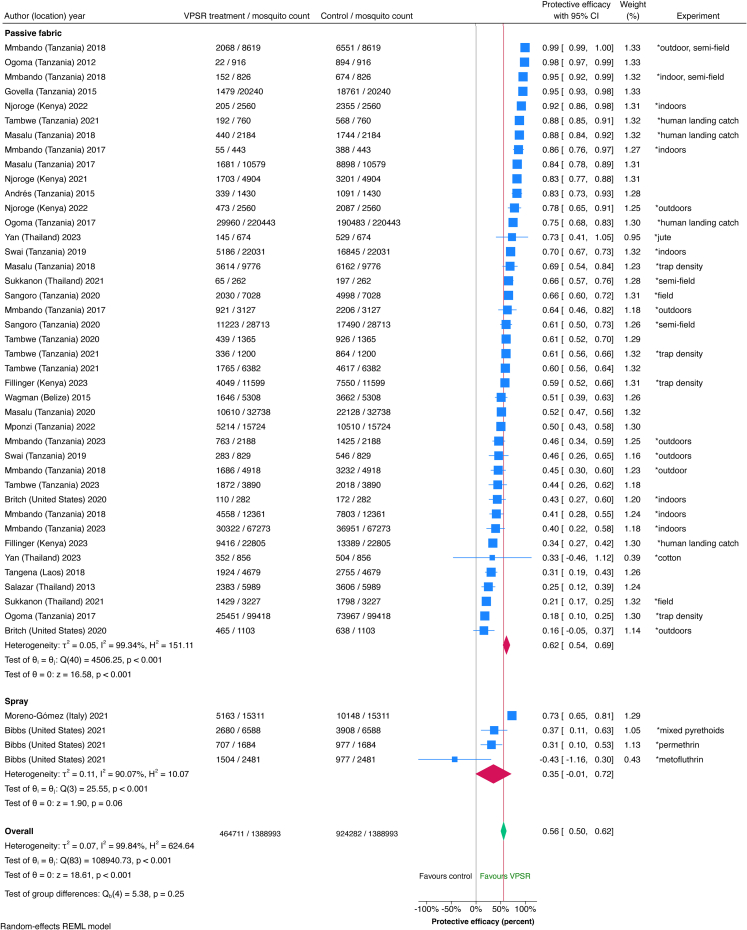
**Study-level forest plot of VPSR protective efficacy subgrouped by intervention format (Part 2).** This figure is divided into two parts, with the first half presented in Fig. 2. Each line represents one study. Average protective efficacy signified by red line based on 1,388,993 mosquitoes (84 studies, combining 39 studies in Fig. 2 and 45 in figure 3). Point estimates are blue boxes sized by weight including confidence intervals (horizontal blue line); exact values for these are provided in the column titled ‘Protective efficacy with 95% CI’. Point estimates to the left of the red line show lower efficacy than average, those to the right have higher efficacy. Some publications had more than one experimental study; the column ‘Experiment’ indicates variables specific to that study. Red diamonds show the average efficacy for each product format. The ‘Weight (%)’ column corresponds to the percentage that each study contributes to the average protective efficacy (total 100% when adding values from figures 2 and 3). The test of θi = θj is a test of homogeneity that uses Cochran's Q test (chi-square), values of p < 0.05 indicates that the studies are statistically different from one another. The test of θ = 0 is a is a z-statistic based on a Wald test that assesses whether overall pooled effects in a group of studies is equal to zero; values of p < 0.05 indicate that overall effects are statistically significantly not equal to zero. These two θ tests are computed for each subgroup. Overall summary statistics of all 84 studies in figures 2 and 3 are provided at the bottom of this figure. For overall pooled estimates, the Q test is a test of group differences where if p < 0.05, the subgroup effect sizes are statistically different from one another. Since p = 0.25, the overall effect sizes from the subgroups are not considered to be different from one another.

Systematic review primary outcomes found a 56% PE (95% CI 50, 62%) overall in a forest plot including 84 studies from 58 publications comprising 1,387,551 mosquitoes (Fig. 2, Fig. 3). Mosquito counts, protective efficacy, and the data source used for the forest plot (publication vs original dataset) are detailed in Appendix Section 2. Subgroups by intervention format showed overlaps in confidence intervals suggesting no statistically significant differences in efficacy between these formats. Heterogeneity was high, with an I^2^ measurement of 99.84%. A summary of the 84 included studies is in Table 3, and details on intervention characteristics, study type, and outcomes measured are in Table 4. Transfluthrin was the most commonly used active ingredient (62% of studies) followed by metofluthrin (28%), mean active ingredient quantities were highest among passive fabric interventions (4197 mg) with coils being the lowest (1.7 mg) and commercial passive devices in between (752 mg). For capture methods, HLC was used in more than half of the studies (59%) particularly those measuring commercial passive devices and passive hessian fabric-based prototypes, while trap density was more often used for all commercial products, some of which were passive while others required energy. For study type, semi-field represented more than half (58%) of studies. Mosquito species varied, with more studies (55) completed on *Anopeheles* mosquitoes compared to *Aedes* (20) and *Culex* (20).

**Table 3:** Summary of intervention formats and study characteristics.

Intervention format	Active ingredients			Quantity of active ingredients (mg)			Capture method		Study type		Mosquito species		
	Metofluthrin	Transfluthrin	Others	Mean^a^	Min	Max	HLC	Traps	Field	Semi-field	Anopheles	Aedes	Culex
Coils n = (13)	7	3	3	1.7	0.036	3.6	4	11	2	11	9	1	7
Commercial passive^b^ n = (18)	10	7	1	752	0.38	3130	12	8	14	3	11	5	4
Passive fabric^b^ n = (35)	0	34	1	4179	0.04	20,000	26	16	15	20	28	8	4
Commercial requiring energy n = (8)	4	1	3	1870	30	3740	1	8	0	8	4	3	5
Sprays n = (4)	1	3	0	4068	40	12,300	3	0	2	3	3	3	0
Total (n = 78)	22	48	8	11,299	75	39,534	46	43	33	45	55	20	20

a Mean active ingredient quantities excluded entries without information (details in Table 2).

b Commercial passive includes any formulated products in development, whether or not they became or will become commercially available. Passive fabric refers to unformulated products prepared by researchers.

**Table 4:** Intervention formats and study characteristics for included studies.

Author (year) (ref)	Country	Study type (field/semi-field)	Intervention format	Study design	Mosquito species	Indoor/outdoor	Active ingredient, producer (if indicated)	Weight (mg)	HLC (yes/no)	Trap density (yes/no)
Lukwa (2008)^24^	Zimbabwe	Field	Coil	Crossover	*An. gambiae*	Outdoor	Metofluthrin	No info	No	Yes
Lukwa (2008)^24^	Zimbabwe	Field	Coil	Crossover	*An. gambiae*	Outdoor	Esbiothrin	No info	No	Yes
Tangena (2018)^25^	Laos	Field	Coil	Latin square	*Ae. albopictus* *An. barbumbrosus* *An. barbirostris* *An. dirus* *An. maculatus* *An. epiroticus* *An. umbrosus* *Cx. vishnui*	Outdoor	Metofluthrin by Fumakilla Ltd, Bangkok, Thailand	1.8	Yes	No
Maia (2016)^26^	Tanzania	Field	Coil	Crossover	*An. arabiensis* *An. funestus* (s.s) *Cx. quinquefasciatus* *Cx. univittatus*	Outdoor	Transfluthrin by SC Johnson and Son	3.6	Yes	Yes
Oumbouke (2017)^27^	Benin	Field	Coil	Latin square	*Cx. quinquefasciatus*	Indoor	Metofluthrin SC Johnson & Son USA	0.75 1.16	No	Yes
Achee (2012)^28^	Thailand	Field	Coil	Crossover	*Aedes aegypti*	Indoor	Metofluthrin SC Johnson & Son USA	0.75	No	Yes
Ogoma (2014)^29^	Tanzania	Semi-field	Coil	Latin square	*An. arabiensis*^a^ *Cx. quinquefasciatus*	Indoor	Transfluthrin	3.6	No	Yes
Syafruddin (2014)^30^	Indonesia	Field	Coil	Randomised controlled trial	*An. sundaicus* *An. subpictus sensu lato* *An. indefinitus* *An. vagus* *An. barbirostris* *An. annularis* *An. maculatus* *An. aconitus* *An. kochi* *An. tessellatus*	Indoor and outdoor	Metofluthrin by SC Johnson Co., Ho Chi Minh, Vietnam	1	Yes	No
Ogoma (2014)^29^	Tanzania	Field	Coil	Latin square	*An. gambiae* s.l. *Cx. Quinquefasciatus*	Indoor	Metofluthrin	0.75	No	Yes
Salazar (2013)^31^	Thailand	Semi-field	Coil	Simultaneous treatment and control assessment	*Ae. aegypti*	Indoor	Metofluthrin S.C. Johnson & Son Inc	0.036 0.078	No	Yes
Vajda (2023)^32^	Thailand	Field	Commercial passive	Crossover	*An. minimus*	Outdoor	Transfluthrin	2000	Yes	No
Zarella (2022)^33^	USA	Field	Commercial passive	Latin square	*Ae. albopictus*	Outdoor	Metofluthrin by Sumimoto chemicals	1200	Yes	No
Kawada (2005)^34^	Indonesia	Field	Commercial passive	Latin square	*An. sundaicus* *Cx. quinquefasciatus*	Outdoor	Metofluthrin by Sumitomo Chemical Co., Ltd	200	Yes	No
McPhatter (2017)^35^	USA	Semi-field	Commercial passive	Randomised controlled trial	*Ae. aegypti*	Outdoor	Transfluthrin by SC Johnson, Racine WI	4.8	No	Yes
Zarella (2022)^33^	USA	Field	Commercial passive	Latin square	*Ae. albopictus*	Indoor	Metofluthrin by Sumimoto chemicals	1200	Yes	No
Swai (2023)^36^	Tanzania	Semi-field	Commercial passive	Crossover	*An. arabiensis*	Indoor	Transfluthrin by SC Johnson & Son	110	Yes	No
Stevenson (2018)^37^	Zambia	Semi-field	Commercial passive	Crossover	*An. gambiae*	Indoor	Metofluthrin	360	No	Yes
Kawada (2008)^38^	Tanzania	Field	Commercial passive	Randomised controlled trial	*An. gambiae*	Indoor	Metofluthrin by Sumimoto Chemicals Company Ltd	600	No	Yes
Devine (2021)^39^	Mexico	Field	Commercial Passive	Randomised controlled trial	*Ae. aegypti*	Indoor	Metofluthrin by Sumimoto Chemicals Company Ltd	1200	Yes	Yes
Charlwood (2014)^40^	Cambodia	Field	Commercial passive	Latin square	*Aedes albopictus*	Outdoor	No info	No info	Yes	No
Charlwood (2016)^41^	Cambodia	Field	Commercial passive	Latin square	*An. minimus* *An. maculates* *An. hodgkini*	Outdoor	Metofluthrin by Sumimoto Chemicals Company Ltd	No info	Yes	Yes
Burton (2023)^42^	Zambia	Semi-field	Commercial passive	Latin square	*An. gambiae*		Transfluthrin by Widder Bros Inc		Yes	No
Charlwood (2017)^43^	Cambodia		Commercial passive	Crossover	*An. dirus*	Indoor	Metofluthrin by Sumimoto Chemicals Company Ltd	1200	No	Yes
Flores-Mendoza (2022)^44^	Peru	Field	Commercial passive	Randomised block	*An. darlingi* *Cx. pedroi* *Cx. corotor*	Outdoor	Metofluthrin	3130	Yes	Yes
Morrison (2022)^45^	Peru	Field	Commercial passive	Randomised controlled trial	*Ae. aegypti*	Indoor	Transfluthrin by SC Johnson & Son	55	No	Yes
Syafruddin (2020)^17^	Indonesia	Field	Commercial passive	Randomised controlled trial	*An. aconitus* *An. annularis* *An. barbirostris* *An. flavirostris* *An. kochi* *An. maculatus* *An. subpictus* *An. sundaicus* *An. tessellatus* *An. vagus*	Indoor	Transfluthrin by SC Johnson & Son	No info	Yes	No
Permana (2022)^46^	Indonesia	Field	Commercial passive	Randomised controlled trial	*An. flavirostris* *An. balabacensis* *An. maculatus* *An. aconitus* *An. kochi* *An. essellatus* *An. sundaicus*	Indoor	Transfluthrin	No info	Yes	No
Permana (2022)^46^	Indonesia	Field	Commercial passive	Randomised controlled trial	*An. flavirostris* *An. balabacensis* *An. maculatus* *An. aconitus* *An. kochi* *An. Essellatus* *An. sundaicus*	Outdoor	Transfluthrin	No info	Yes	No
Syafruddin (2020)^17^	Indonesia	Field	Commercial passive	Randomised controlled trial	*An. aconitus* *An. annularis* *An. barbirostris* *An. flavirostris* *An. kochi* *An. maculatus* *An. subpictus* *An. sundaicus* *An. tessellatus* *An. vagus*	Outdoor	Transfluthrin by SC Johnson & Son	No info	Yes	No
Kawada (2008)^38^	Tanzania	Field	Commercial passive	Randomised controlled	*An. gambiae*	Indoor	Metofluthrin by Sumimoto Chemicals Company Ltd	600	No	Yes
Stevenson (2018)^37^	Zambia	Semi-field	Commercial passive	Crossover	*An. gambiae*	Outdoor	Metofluthrin	360	No	Yes
Lloyd (2013)^47^	USA	Field	Commercial product requiring energy	Crossover	*Ae. albopictus*	Outdoor	Allethrin	No info	No	Yes
Lloyd (2013)^47^	USA	Field	Commercial Product Requiring Energy	Crossover	*Ae. albopictus*	Outdoor	Metofluthrin by SC Johnson & Son	No info	No	Yes
Britch (2021)^48^	USA	Field	Commercial product requiring energy	Latin square	*An. quad-rimaculatus* *Cx. erraticus*	Outdoor	Metofluthrin by SC Johnson & Son	3740	No	Yes
Frances (2020)^49^	Australia	Field	Commercial product requiring energy	Randomised controlled	*Aedes vigilax*	Outdoor	Metofluthrin by SC Johnson, Sydney, Australia	3740	No	Yes
Britch (2021)^48^	USA	Field	Commercial product requiring energy	Latin square	*An. quad-rimaculatus* *Cx. erraticus*	Outdoor	Allethrin by Schwabel Corp	2600	No	Yes
Dame (2014)^50^	USA	Field	Commercial product requiring energy	Latin square	*An. quadrimac-ulatus* *Cx. erraticus*	Outdoor	Metofluthrin by SC Johnson & Son	3740	No	Yes
Dame (2014)^50^	USA	Field	Commercial product requiring energy	Latin square	*An. quadrimac-ulatus* *Cx. erraticus*	Outdoor	Allethrin by Schwabel Corporation	2600	No	Yes
Mmbando (2018)^51^	Tanzania	Semi-field	Passive fabric	Treatment vs control	*An. arabiensis*	Outdoor	Transfluthrin by Shenzhen Sunrising Industry	600	Yes	Yes
Ogoma (2012)^52^	Tanzania	Semi-field	Passive fabric	Latin square	*An. arabiensis*	Outdoor	Transfluthrin by SC Johnson & Son	25	Yes	No
Mmbando (2018)^51^	Tanzania	Semi-field	Passive fabric	Treatment vs control	*An. gambiae* *An. funestus*	Indoor	Transfluthrin by Shenzhen Sunrising Industry	180	No	Yes
Govella (2015)^53^	Tanzania	Field	Passive fabric	Crossover	*An. gambiae*	Outdoor	Transfluthrin by Shenzhen Sunrising Industry Company	10,000	Yes	No
Njoroge (2022)^54^	Kenya	Semi-field	Passive fabric	Latin square	*An. arabiensis*	Indoor	Transfluthrin by Bayer Global	200	Yes	No
Tambwe (2021)^55^	Tanzania	Semi-field	Passive fabric	Crossover	*Ae. aegypti*	Outdoor	Transfluthrin	5205	Yes	No
Masalu (2018)^56^	Tanzania	Field	Passive fabric	Latin square	*An. arabiensis* *An. funestus* *Cx.* spp.	Indoor	Transfluthrin by Shenzhen Sunrising Industry Company	5000	Yes	No
Mmbando (2017)^57^	Tanzania	Field	Passive fabric	Crossover	*An. gambiae* s.l. *An. funestus* s.s.^a^	Indoor	Transfluthrin by A-to-Z textile mills Ltd, Arusha Tanzania	170	Yes	Yes
Masalu (2017)^58^	Tanzania	Field	Passive fabric	Latin square	*An. arabiensis* *Cx.* spp.	Outdoor	Transfluthrin	5000	Yes	No
Njoroge (2021)^59^	Kenya	Semi-field	Passive fabric	Latin square	*An. arabiensis*	Indoor	Transfluthrin by Bayer Global, Leverkusen, Germany	200	Yes	Yes
Andrës (2015)^60^	Tanzania	Semi-field	Passive fabric	Crossover	*An. arabiensis* *An. gambiae*	Outdoor	Transfluthrin	90	Yes	No
Njoroge (2022)^54^	Kenya	Semi-field	Passive fabric	Latin square	*An. arabiensis*	Outdoor	Transfluthrin by Bayer Global	200	Yes	No
Ogoma (2017)^61^	Tanzania	Field	Passive fabric	Latin square	*An. Arabiensis* *An. funestus* *An. coustani*	Outdoor	Transfluthrin by Shenzhen Sunrising Industry Company	5000	Yes	Yes
Yan (2023)^62^	Thailand	Semi-field	Passive fabric	Randomised controlled	*An. minimus*	Indoor	Transfluthrin	2400	Yes	No
Swai (2019)^63^	Tanzania	Field	Passive fabric	Latin square	*An. arabiensis* *An. funestus*	Indoor	Transfluthrin	180	No	Yes
Masalu (2018)^56^	Tanzania	Field	Passive fabric	Latin square	*An. arabiensis* *An. funestus* *Cx.* spp.	Indoor	Transfluthrin by Shenzhen Sunrising Industry Company	5000	Yes	No
Sukkanon (2021)^64^	Thailand	Semi-field	Passive fabric	Crossover (rotated collectors)	*An. harrisoni*	Indoor	Transfluthrin	0.8	Yes	No
Sangoro (2020)^65^	Tanzania	Field	Passive fabric	Crossover	*An. funestus* *An. arabiensis*	Outdoor	SC Johnson & Son	150	Yes	Yes
Mmbando (2017)^57^	Tanzania	Field	Passive fabric	Crossover	*An. gambiae* s.l. *An. funestus* s.s.^a^	Outdoor	Transfluthrin by A-to-Z textile mills Ltd, Arusha Tanzania	170	Yes	Yes
Sangoro (2020)^65^	Tanzania	Semi-field	Passive fabric	Crossover	*An. funestus* *An. arabiensis*	Outdoor	Transfluthrin by SC Johnson & Son	150	Yes	Yes
Tambwe (2020)^66^	Tanzania	Semi-field	Passive fabric	Crossover	*Ae. aegypti*	Outdoor	Transfluthrin by SC Johnson Home Hygiene Products	5250	Yes	Yes
Tambwe (2021)^55^	Tanzania	Semi-field	Passive fabric	Latin square	*Ae. aegypti*	Outdoor	Transfluthrin	5205	Yes	No
Tambwe (2021)^67^	Tanzania	Semi-field	Passive fabric	Latin square	*An. gambiae* (Ifakara strain) *An. arabiensis* (Mbita strain) *An. arabiensis* (Kingani strain)	Outdoor	Transfluthrin by Bayothrin EC, Bayer AG, Germany	5250	Yes	No
Fillinger (2023)^68^	Kenya	Field	Passive fabric	Randomised controlled	*An. funestus* *An. arabiensis* *Cx. mansonia*	Indoor and outdoor	Transfluthrin	2500	Yes	Yes
Wagman (2015)^69^	Belize	Field	Passive fabric	Latin square	*An. vestitipennis* *An. albimanus*	Outdoor	Transfluthrin by SC Johnson and Son, Inc	42.3	No	Yes
Masalu (2020)^70^	Tanzania	Field	Passive fabric	Latin square	*An. arabiensis* *An. funestus*	Outdoor	Transfluthrin by Bayer AG, Germany	240	No	Yes
Mponzi (2022)^71^	Tanzania	Semi-field	others	Crossover	*Ae. aegypti* *An. arabiensis*	Indoor	Transfluthrin	40	Yes	No
Mmbando (2023)^72^	Tanzania	Field	Passive fabric	Randomised controlled	*An. arabiensis* *An. funestus*	Outdoor	Transfluthrin by A-to-Z Textile Mills Ltd, Arusha, Tanzania	350	No	Yes
Swai (2019)^63^	Tanzania	Field	Passive fabric	Latin square	*An. arabiensis* *An. funestus*	Outdoor	Transfluthrin	300	No	Yes
Mmbando (2018)^51^	Tanzania	Semi-field	Passive fabric	Treatment vs control	*An. gambiae* *An. funestus*	Indoor	Transfluthrin by Shenzhen Sunrising Industry	180	No	Yes
Tambwe (2023)^73^	Tanzania	Semi-field	Passive fabric	Crossover	*An. gambiae* *An. funestus*	Outdoor	Transfluthrin by Bayothrin EC; Bayer, Monheim am Rhein, Germany	5000 10,000 15,000 20,000	Yes	No
Britch (2020)^74^	USA	Field	Passive fabric	Treatment vs control	*Aedes dorsalis* *An. hermsi* *Culiseta inorta* *Cx. erythrothorax* *Cx. quinquefasciatus* *Cx. tarsalis*	Indoor	Transfluthrin	8000	No	Yes
Mmbando (2018)^51^	Tanzania	Semi-field	Passive fabric	Treatment vs control	*An. arabiensis*	Indoor	Transfluthrin by Shenzhen Sunrising Industry	600	Yes	Yes
Mmbando (2023)^72^	Tanzania	Field	Passive fabric	Randomised controlled	*An. arabiensis* *An. funestus*	Outdoor	Transfluthrin by A-to-Z Textile Mills Ltd, Arusha, Tanzania	350	No	Yes
Fillinger (2023)^68^	Kenya	Field	Passive fabric	Randomised controlled	*An. funestus* *An. arabiensis* *Cx. mansonia*	Indoor and outdoor	Transfluthrin	2500	Yes	Yes
Yan (2023)^62^	Thailand	Semi-field	Passive fabric	Randomised controlled	*An. minimus*	Indoor	Transfluthrin	2400	Yes	No
Tangena (2018)^25^	Laos	Field	Passive fabric	Latin square	*Ae. albopictus* *An. barbumbrosus* *An. barbirostris* *An. dirus* *An. maculatus* *An. epiroticus* *An. umbrosus* *Cx. vishnui*	Outdoor	Metofluthrin by Fumakilla Ltd, Bangkok, Thailand	1.8	Yes	No
Salazar (2013)^31^	Thailand	Semi-field	Passive fabric	Simultaneous treatment and control assessment	*Ae. aegypti*	Indoor	Transfluthrin by Bayer, AG	0.04	No	Yes
Sukkanon (2021)^64^	Thailand	Field	Passive fabric	Crossover (rotated collectors)	*An. minimus* *Aedes* spp.	Outdoor	Transfluthrin	0.8	Yes	No
Ogoma (2017)^61^	Tanzania	Field	Passive fabric	Latin square	*An. Arabiensis* *An. funestus* *An. coustani*	Outdoor	Transfluthrin by Shenzhen Sunrising Industry Company	5000	Yes	Yes
Britch (2020)^74^	USA	Field	Passive fabric	Treatment vs control	*Ae. dorsalis* *An. hermsi* *Cu. inorta* *Cx. erythrothorax* *Cx. quinquefasciatus* *Cx. tarsalis*	Outdoor	Transfluthrin	8000	No	Yes
Moreno-Gómez (2021)^75^	Italy	Field	Spray	Crossover	*Ae. albopictus*	Outdoor	Transfluthrin by Henkel's chemical laboratory in San Marino	12,300	Yes	No
Bibbs (2021)^76^	USA	Field	Spray	Treatment vs control	*Ae. albopictus*	Outdoor	Mixed pyrethroids	3840	No	Yes
Bibbs (2021)^76^	USA	Field	Spray		*Ae. albopictus*	Outdoor	Permethrin	3840	No	Yes
Bibbs (2021)^76^	USA	Field	Spray		*Ae. albopictus*	Outdoor	Metofluthrin	3840	No	Yes
Pates (2002)^77^	Tanzania	Field	Commercial requiring energy	Latin square	*Cx. quinquefasciatus*	Outdoor	Transfluthrin	151 151 30 75	Yes	No
Kitau (2010)^78^	Tanzania	Semi-field	Commercial passive	Latin square	*Cx. Quinquefasciatus* *An. gambiae*	Indoor	d-allethrin	25	Yes	Yes
Msangi (2010)^79^	Tanzania	Field	Coil	Latin square	*Cx. quinquefasciatus* *An. gambiae*	Indoor	d-allethrin Pyrethrin	2.3 3 3 1.5	No	Yes
Rapley (2009)^80^	Australia	Semi-field	Commercial passive	Crossover	*Ae. aegypti*	Indoor	Metofluthrin	26	Yes	No
Rapley (2009)^80^	Australia	Semi-field	Commercial passive	Crossover	*Ae. aegypti*	Indoor	Metofluthrin	26	Yes	No

a Information is from personal correspondence.

Secondary entomological outcomes assessed are in Table 5; most commonly knock-down and mortality, collected in 27% and 36% of the 78 studies respectively, the majority of which were studies on coils. Blood feeding inhibition was collected as a proportion in 17% of studies, and the number of blood fed mosquitoes in another 10% of studies, mostly on commercial passive and passive fabric interventions. Deterrence was also measured in 14% of studies, while fecundity reduction was only measured in one study. No studies included investigated non-contact irritancy.

**Table 5:** Secondary entomological outcomes measured.

Intervention format	Study type		Entomological outcomes measured (number of studies)						
	Semi-field	Field	Knockdown	Mortality	Blood-feeding inhibition		Deterrence	Fecundity reduction	Non-contact irritancy
					Proportion	No. blood fed			
Coil	2	11	9	10	3	0	3	0	0
Commercial passive	14	3	6	7	5	5	4	0	0
Passive fabric	15	20	2	7	5	3	3	0	0
Commercial requiring energy	0	8	1	1	0	0	1	0	0
Spray	2	3	3	3	0	0	0	1	0
Total	33	76	21	28	13	8	11	1	0

Study-level findings and methodological details are in Table S1. Insecticide resistance data are summarised in Table 6, with study-level details in Table S2, showing that wild *An. arabiensis* and *An. funestus* mosquitoes in east Africa had high levels of solid-state pyrethroid resistance, while *Aedes*, *An. gambiae* s.s., and various *Anopheles* mosquito species observed in Southeast Asia showed mixed susceptibility to solid-state pyrethroids. *Culex* mosquitoes showed limited data available although those that measured susceptibility showed high levels of resistance to pyrethroids.

**Table 6:** Pyrethroid resistance.

Vector species	Pyrethroid resistance^a^ (number of studies)			
	Confirmed resistance	Possible resistance	Confirmed susceptible	No information on resistance
**Dengue global**				
*Ae. aegypti*	3	0	5	5
*Ae. albopictus*	1	1	5	0
*Ae.* other	0	0	0	1
**Malaria Latin America**				
*An. darlingi* complex	0	1	1	0
*An.* other	0	2	0	0
**Malaria Southeast Asia**				
*An. dirus* complex	0	0	0	1
*An. farauti* complex	0	0	0	0
*An. minimus* complex	0	2	1	0
*An.* other	6	4	1	6
**Malaria East and Southern Africa**				
*An. gambiae* s.s.	4	0	4	3
*An. arabiensis*	17	2	0	0
*An. funestus*	10	0	0	0
*An.* other	0	0	0	1
**Malaria West Africa (no data)**				
**Nuisance biting global**				
*Culex*	3	0	0	7
U.S. species	0	0	1	8

a For species with different resistance profiles to various pyrethroids, the category with strongest resistance was selected.

**Fig. 4:**
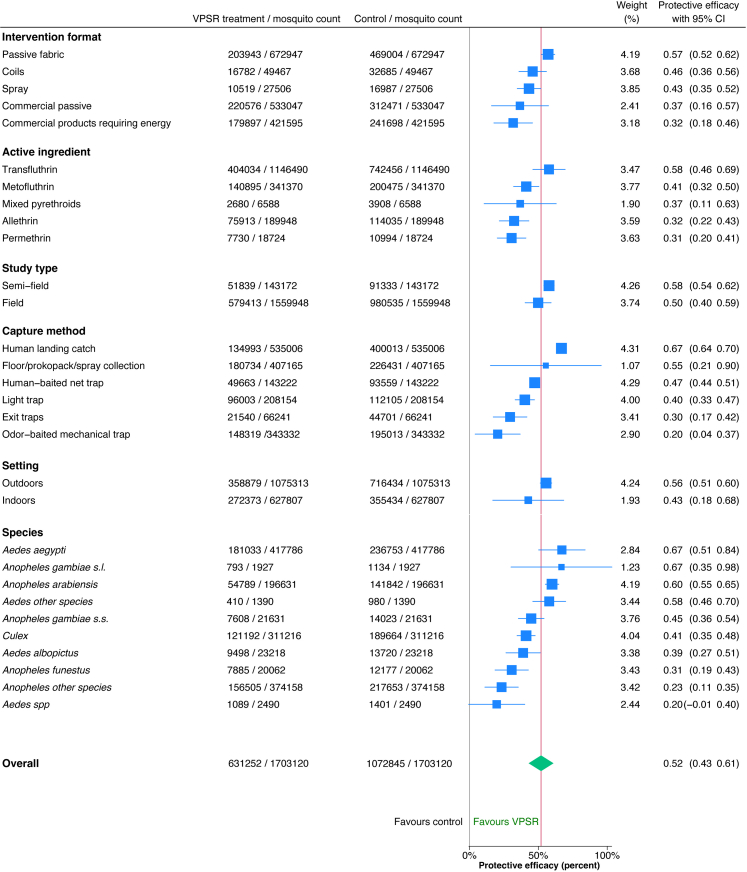
**Pooled meta-analysis of VPSR efficacy subgrouped by select parameters.** Combined standardised individual mosquito-level data of 1,703,120 mosquitoes from 50 studies analysed by subgroups; red line represents average protective efficacy, with efficacy to the right signifying higher efficacy, and lower efficacy to the left. Pooled estimates in blue boxes sized by weight, with horizontal blue lines indicating confidence intervals.

For the pooled meta-analysis, individual mosquito-level data from 1,703,120 mosquitoes were combined from 50 studies, providing an average PE of 52% (95% CI 43, 61%) (Fig. 4). Details on each variable and corresponding coding parameters are tabulated in Appendix Section 3. For intervention formats, passive fabric had higher PE (57%, 95% CI 52, 62%) compared to commercial products requiring energy (32%, 95% CI 18, 46%), while other formats showed no statistically significant differences in efficacy based on overlapping confidence intervals. For active ingredients, transfluthrin (58% PE, 95% CI 44, 68%) and metofluthrin (41% PE, 95% CI 31, 49%) were the most efficacious. For capture methods, HLC showed higher PE (67%, 95% CI 64, 70%) than traps. For intervention format, passive fabric formats showed highest PE (57%, 95% CI 52, 62%), and that of commercial passive devices had a wide confidence interval (37%, 95% CI 16, 57%). For study types, semi-field studies showed slightly higher efficacy than field studies although this was not statistically different (58% PE, 95% CI 54, 62% for semi-field, 50%, 95% CI 40, 59% for field). For indoor versus outdoor settings, these could not be compared because the confidence interval for indoor studies was wide (43%, 95% CI 11, 63%). Significant differences in PE were seen across various mosquito species; the highest was for *An. arabiensis* (75%, 95% CI 72, 78%) with *An. gambiae* *s.s.* having average PE (53%, 95% CI 47, 59%), *Culex* below average (41% PE, 95% CI 34, 47%), and *An. funestus* having low PE (31%, 95% CI 18, 42%). Other *Anopheles* and *Aedes* species measured showed wide PE confidence intervals. The potential for cross-resistance between solid-state pyrethroids and VPSRs is unclear; *An. funestus* studies were conducted in Tanzania and Kenya where mosquito populations have high levels of insecticide resistance.

**Fig. 5:**
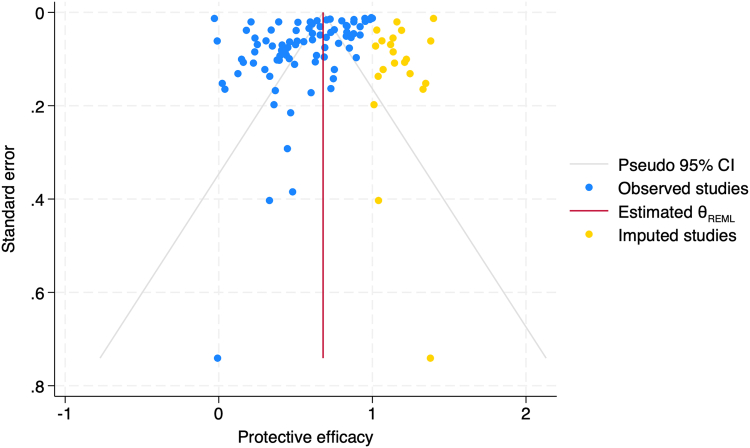
**Funnel plot to assess for publication bias.** The blue dots each correspond to a study in the forest plots in Fig. 2, Fig. 3 (84 studies total), the red line is the overall effect (56%), which uses a Restricted Maximum Likelihood (REML) estimate indicating its precision through overall effect size using confidence intervals after controlling for within and between study variability. The included studies shown in blue are mostly between the confidence intervals indicated by grey lines in the shape of an upside-down funnel, suggesting there is no publication bias. Imputed studies shown in yellow estimate the results of missing studies that would address publication bias if it was present; since these have higher protective efficacy of volatile pyrethroid spatial repellents than published studies, publication bias is unlikely.

Sensitivity analysis found no statistically significant impact of temperature, humidity, and wind on overall PE for outdoor studies, and no significant impact on overall PE estimates when dropping one study at a time (see Appendix Section 4). RoB was found to be low or unclear for most categories (Table 7; heat map of study-level results in Table S3). Study design bias was low because we required for included studies to control for spatial and temporal heterogeneity. Study participants, investigators, and statisticians were often not blinded, resulting in unclear risk of bias. Selective outcome reporting was low, and mosquito semi-field conditions were mostly assessed as having low RoB. For funder involvement, three studies were assessed with concern of RoB because funders were co-authors on the studies. Publication bias was unlikely based on a funnel plot (Fig. 5), results of which suggest that this review may be missing studies showing higher PE of VPSRs. Publication bias is therefore unlikely, with these findings likely reflecting the inherent heterogeneity of this evidence base. Begg's test statistic to detect publication bias was also not significant (p = 0.1382).

**Table 7:** Risk of bias summary.^a^

Risk of bias	Randomisation for spatial and temporal heterogeneity		Blinding				Mosquito conditions (semi-field studies only)	Funder involvement
	Study design	Cluster RCTs only: baseline characteristics	Participants	Investigator	Statistician	Outcome reporting		
High	–	–	–	–	–	–	–	–
Some concern	–	–	–	–	–	–	–	3 (5%)
Low	58 (100%)	3 (100%)	10 (17%)	2 (3%)	3 (5%)	51 (88%)	18 (72%)	30 (52%)
Unclear	–	–	48 (83%)	56 (97%)	53 (95%)	7 (12%)	7 (28%)	25 (43%)

a Numbers signify number of publications from forest plot (n = 58 total), with percentages for each column adding up to 100%.

Our dates for inclusion for systematic review and meta-analysis do not include nine studies published between September 7, 2023 and July 28, 2025. These are summarised in Table 8; from West Africa, a field study on the use of a transfluthrin-based commercial product in Benin was published in 2024, finding 34% efficacy (95% CI 22, 44%).^81^ Field studies have been conducted in Haiti, Kenya, Cambodia, Indonesia, coastal Tanzania, and Florida in USA, showing mixed results ranging from no statistically significant results (Haiti,^83^ the USA,^82^ and coastal Tanzania^85^) to PEs 94% and above in Cambodia^87^ and Kenya.^86^ Two semi-field studies show efficacy both for passive fabric prototypes in Tanzania (PE 50–60%)^85^ and for the transfluthrin-based BiteBarrier® product (PE 80–98%).^84^ A randomised controlled trial in Kenya evaluated the epidemiological impact of a VPSR but was not powered to detect entomological endpoints, and no significant intervention effect was found based on this entomological substudy.^18^

**Table 8:** Studies published between September 7th, 2023 and July 28th, 2025.

Author (country) year (citation)	Study details	Findings
Fongnikin (Benin) 2024^81^	Field study using HLC to assess commercial transfluthrin-based product Mosquito Shield™ compared to placebo	PE of 34.2% (22.1 to 44.4%, p < 0.001) against wild pyrethroid-resistant *An. gambiae* s.l. mosquitoes where PE = (1−IRR) ∗ 100, IRR is the incidence rate ratio in the Mosquito Shield ™ group.
Bayer (United States) 2024^82^	Field study using CDC light traps to assess transfluthrin-impregnated devices lasting 150 or 250 days versus control in military tents	No statistically significant differences in trap density seen compared to control, although higher numbers of mosquitoes and mortality were observed in tents with transfluthrin during the first two weeks of study. Five main species of mosquitoes were found; *Cx. Nigripalpus*, *An. crucians*, *Cx. Erraticus*, *Coquillettidia perturbans*, and *Ae. Tormentor*. A general linear model with family quasi-Poisson was generated, and mean collection numbers were evaluated by Ryan–Einot–Bagriel–Welsch multiple range test to assess for differences between the number of mosquitoes collected in treated tents vs control.
Supreme (Haiti) 2024^83^	Field study using HLC to assess transfluthrin-treated fabric versus control in urban areas	No statistically significant differences seen in landing rates of wild *Ae. Aegypti* mosquitoes, with 437 females caught over 985 h of collection (relative landing rate 0.87, 95% CI 0.73, 1.04, p = 0.1241). Generalised linear mixed models were fitted to longitudinal datasets to account for effects of spatiotemporal variations in mosquito density, with random effects including date, time of day, and collection station.
Vajda (Thailand) 2024^84^	Semi-field study using HLC to assess transfluthrin (Bitebarrier® new, aged 20 and aged 30 days, Fuyi Sin spray) and metofluthrin (SumiOne® new) versus control	PE of new BiteBarrier® was 80 to 98% (95% CI 71, 99%) in two sites across two replicates, with similar results seen in devices aged up to 30 days. This was 84 to 95% for Fuyi Sin (95% CI 71, 97%), and 91 to 93% (95% CI 81, 96) for SumiOne® against pyrethroid-susceptible *An. minimus* mosquitoes. Reductions in post-exposure blood feeding, induced knockdown, and 24 h mortality were observed for all VPSRs tested. Intervention effects were estimated on dichotomous entomology endpoints compared to control as odds ratios estimated using logistic regression analysis with intervention as a fixed effect, and batch effect (clustering of mosquitoes within the same chamber-night) as a random effect.
Govella (Tanzania) 2024^85^	Semi-field and field study using HLC to assess transfluthrin-treated fabric versus control in coastal areas	PE of 50 to 60% observed in semi-field studies using wild-caught *Ae. Aegypti* as well as lab-reared susceptible *Ae. Aegypti* and *An. gambiae* mosquitoes. Negligible reductions observed in field conditions. A generalised linear mixed model was fitted to data collected at temperatures above 22 °C, assuming a Poisson distribution of data with date treated as a random effect.
Agumba (Kenya) 2024^86^	Field study using HLC to assess metofluthrin vapouriser using liquid petroleum gas canister by Thermacell Repellents, Inc in experimental huts or outdoors versus control	PE of 99.3% observed against wild *An. funestus* mosquitoes, with knockdown rate of 95.5% and mortality of 97.7% in treated huts. In the outdoor study, landing rate was significantly lower for all distances from the emanator, with larger effects seen at 5 feet (RR = 0.151; 95% CI [0.070–0.327]; p < 0.001) and 10 feet (RR = 0.063; 95% CI [0.021–0.192]; p < 0.001), as compared to 20 feet from the emanator (RR = 0.547; 95% CI [0.331–0.905]; p = 0.019). Generalised linear mixed models were fitted using negative binomial distribution for analysis of mosquito numbers, adjusted for random effects such as repeated measures using the hut, compound identification, and hour.
Vajda (Cambodia) 2024^87^	Field study using HLC to assess commercial transfluthrin-based product BiteBarrier® in temporary open structures vs control	PE of 94% (95% CI 93, 96% p < 0.001) observed against wild *Anopheles* mosquitoes, 96% of which were *An. dirus*. PE was estimated as (1−RR) ∗ 100, RR is the rate ratio on the number of *Anopheles* mosquitoes landed in the intervention arm compared to that of control.
Burton (Indonesia) 2025^88^	Field study using HLC to assess commercial transfluthrin-based product BiteBarrier® in temporary shelters versus control	Relative rate of reduced host seeking for VPSR of 0.30 [0.21–0.43], p < 0.001 observed for exposed *Anopheles* mosquitoes. PE affected by collection locations and device age over two weeks; these impacts on device age were not seen in other studies on this product. Generalised linear models were generated for nightly biting rates linked with a negative binomial distribution after confirming for overdispersion in a Poisson-distributed model, with collection date as a random effect and location, device age, collector, and weather variables as fixed effects.
Burton (Indonesia 2) 2025^89^	Field study using double-net traps to assess commercial transfluthrin-based product BiteBarrier® in open-walled houses versus control	Relative rate of reduced host seeking for VPSR of 0.29 [0.19–0.45], p < 0.001 observed for exposed *Anopheles* mosquitoes, with 1 month age of product not impacting modelled efficacy. A generalised linear mixed effect model was used with fixed effects including socioeconomic status, age, spatial and weather variables, and random effects including collection date and cluster nested within location.

## Discussion

In this systematic review and meta-analysis of the entomological PE of VPSRs, we evaluated mosquito landing behaviour among 58 publications from 16 countries completed between 2000 and September 2023, finding a mean PE of 56% (95% CI 50, 62%) in the systematic review. Mosquito-level data was made available for 50 (86%) of these studies comprising 1,703,120 mosquitoes showing a pooled PE of 52% (95% CI 43, 61%). VPSRs were efficacious with highest PE seen in passive fabric formats (57% PE, 95% CI 52, 62%) using transfluthrin, although this format also used highest doses of active ingredients (average 4179 mg). For coils, efficacy was also high (46% PE, 95% CI 36, 56%) despite short durations of efficacy. Although commercial passive products were efficacious, these had a wide confidence interval (37% PE, 95% CI 16, 57%) revealing the need for more evaluations, particularly in outdoor field settings. For nine publications made available between September 7, 2023 and July 28, 2025, we summarised study objectives and findings, most (7/9) of which used HLC as an endpoint. These findings provide insights into VPSR testing methods, rollout, evidence gaps, and future research needed that could support the VPSR WHO policy recommendation^15^ and guide the harmonisation of global regulatory assessments.^19^

For VPSR testing methods, our study shows that these have evolved substantially over the past 20 years, and that future studies may benefit from standardising methods in a process similar to that undertaken for ITN evaluation.^90^ We confirm that mosquito landing inhibition is best assessed using the gold standard HLC (67% efficacy, 95% CI 64, 69%).^73^ Our results also confirm that semi-field study designs, which are recommended by the WHO as part of the spatial repellent evaluation process,^19^ are suitable for VPSR evaluation as they provide similar results to those in field settings (58%, 95% CI 54, 62% vs 50%, 95% CI 40, 58% respectively). Semi-field studies offer an additional advantage of collecting multiple entomological endpoints safely and systematically in a high-throughput manner at considerably less cost than field trials.^91^

For entomological outcomes, we recommend that landing inhibition or feeding inhibition remain a primary outcome, and that additional outcomes also be measured as these may also contribute to a reduction in disease transmission that can be estimated using mathematical models.^91^^,^^92^ Our review shows that the most commonly secondary outcomes were knockdown and mortality, confirming that VPSRs can confer both individual and community-level protection. More data is needed on VPSR sublethal effects. Outcomes in this review include deterrence and non-contact irritancy from experimental huts,^36^ diversion to other hosts (for which one study suggests is unlikely to occur beyond a distance of 10 m),^55^ and fecundity to infer downstream reductions of mosquito populations. Disarming, defined as sublethal incapacitation for one night,^84^ can also be used in future studies as a measure of community-level bite reduction.

Mathematical models of the impact of VPSRs on disease transmission should examine the use of VPSR products alone where ITNs cannot be deployed, as well as when used with ITNs and other interventions, exploring different transmission and vector ecotype settings to identify minimum VPSR coverage levels needed to reduce disease transmission.^91^ Since commercial products have different active ingredient release rates and durability, each product should be modelled individually to identify optimal use case scenarios for specific to that product.

When selecting a VPSR product for rollout, our study confirms that passive products using transfluthrin or metofluthrin are efficacious. While passive fabric interventions showed the highest efficacy, this might be driven by dose response as these contained up to 20 g of transfluthrin and had high surface area. We do not recommend the use of these passive fabric interventions, as they lack formal safety assessments while commercial products are required to undergo rigorous safety assessments, containing lower concentrations of active ingredients (up to 4 g) formulated for slow release. For product selection, durability is an important factor that was not assessed in our study; as users are more likely to adopt and adhere to interventions that offer long-term efficacy with minimal compliance or maintenance requirements.^11^ Coils last for 8 to 9 h while passive commercially available products included in this study have approximately a month of efficacy.^36^^,^^42^ Passive treated fabric interventions often show months of efficacy with some studies demonstrating more than one year of efficacy but, as discussed above, contain high doses of active ingredient and are not confirmed to be safe.^61^ Product durability should be selected based on the use case. A product lasting a few weeks may be sufficient for temporary use cases (e.g., military assignments, travel) while for everyday use, longer-lasting products will be more suited to public health applications, minimising the need for compliance,^11^ frequent distribution, and replacement, increasing user acceptability^93^ and cost-effectiveness.^94^ The longest lasting commercial product available is a new product called SC Johnson Guardian™, which has similar PE to other VPSRs including SC Johnson's Shield™ but has more than 12 months of efficacy in indoor experimental hut studies.^95^

For targeting rollout locations and use case scenarios, VPSRs showed different levels of efficacy across species of mosquitoes, a finding similar to studies on topical repellents^96^ and ITNs.^97^ As mosquito species and their disease transmission potential vary by location,^98^ we recommend that VPSR rollout to prevent dengue and malaria consider local vector species, human behaviour, and existing interventions to ensure that these products address protection gaps during peak biting times.^8^ Table 9 provides a framework for this considering ecotype,^99^ vector presence and binomics,^100^ and pyrethroid resistance^101^ in specific geographical settings, revealing a need for more VPSR data on *An. farauti*, the major vector species in Papua New Guinea, *An. dirus*, an outdoor-biting vector in Southeast Asia, and *An. stephensi*, a generally urban vector spreading rapidly in Africa. Use case scenarios within these settings include peri-domestic and indoor use outside of sleeping hours when ITNs are available,^102^ and protection for high-risk groups and situations when ITNs are not available^103^ including protection for mobile workers,^103^ refugees and workers during humanitarian crises,^104^ military personnel, and tourists in disease-endemic areas. Our study confirms that in tropical conditions, the combined effects of temperature, humidity, and wind, were not associated with outdoor PE, allowing VPSRs to remain efficacious despite likely temporary changes to the size and shape of the bubble of protection offered by VPSRs caused by climatic conditions.

**Table 9:** Framework for VPSR use based on ecotype and vector presence in specific geographical settings.

Location	Dengue	Malaria						
	Global	South Asia, the Middle East, and North Africa	Latin America	Southeast Asia	East and Southern Africa		West Africa	
Ecotype	Tropical or subtropical urban	Tropical or subtropical urban	Tropical or subtropical moist forest/plantations	Tropical or subtropical moist forest/plantations	Tropical or subtropical savannah/arable	Tropical or subtropical moist forest/arable	Tropical or subtropical savannah/arable	Tropical or subtropical moist forest/arable
Main vector	*Ae. aegypti* *Ae. albopictus*	*An. stephensi*	*An. darlingi* complex	*An. dirus* complex *An. farauti* complex *An. minimus* complex	*An. arabiensis* *An. funestus*	*An. gambiae* s.s. *An. arabiensis* *An. funestus*	*An. arabiensis* *An. funestus*	*An. gambiae* s.s. *An. arabiensis* *An. funestus*
Vector biting time	Day	Crepuscular	Crepuscular	Crepuscular	Mainly night, some crepuscular	Mainly night, some crepuscular	Mainly night, some crepuscular	Mainly night, some crepuscular
Vector biting location	Indoors and outdoors	Outdoors	Outdoors	Outdoors	Mainly indoors and some outdoors	Mainly indoors and some outdoors	Mainly indoors and some outdoors	Mainly indoors and some outdoors
Preference for feeding on humans	High	High unless cattle available	Medium	Medium	High (*An. arabiensis* will preferentially feed on cattle when present)	High (*An. arabiensis* will preferentially feed on cattle when present)	High (*An. arabiensis* will preferentially feed on cattle when present)	High (*An. arabiensis* will preferentially feed on cattle when present)
Pyrethroid resistance	Medium	Low	Susceptible	Susceptible	High	High	Very high	Very high

For deployment, practical and low-cost methods that monitor continued product efficacy over time are urgently needed to determine product durability. When products are rolled out, implementation science research can assess and support product uptake among end-users^93^ and should include community integration and engagement activities,^105^ targeted social and behaviour change communication strategies,^106^ and cost-effectiveness analysis. Concurrent to VPSR deployment we need to recognise that the use of passive VPSRs as a single active ingredient chemical class will select for insecticide resistance.^107^ The identification and development of new active ingredients is an urgent priority as the vector control product development process has high attrition rates and long regulatory timelines.

For evidence gaps and future research, our study reveals the need for more studies on commercial products, for which we recommend a focus on field studies of outdoor use case scenarios described above. For research locations, more data are needed from West African, South American, and Southeast Asian settings, where the burden of mosquito-borne disease is high and evidence from field data is scarce. Methodological research to investigate mosquito exposure-free methods for evaluating landing inhibition would be extremely valuable as such procedures could replace HLCs, which carry risks of contracting vector-borne disease in field settings. The human baited double-net trap may be a useful replacement for HLC.^32^ For mosquito species, more studies are needed on *Aedes* mosquitoes to help address the growing burden of dengue fever.^45^ More data on how insecticide resistance affects VPSR efficacy are essential. Future studies can use the WHO's recent guidance on transfluthrin discriminating concentrations to measure for the impact of resistance^108^^,^^109^ to monitor for potential cross-resistance with solid-state pyrethroids and other insecticides. Collaborating institutions can also conduct durability and resistance monitoring to ensure the uniformity of chemical dilutions and evaluation procedures.

Our study has several strengths and limitations. The main strength is the quantity of data collected across published studies and high level of data availability for meta-analysis, providing insights on VPSR efficacy as well as factors affecting efficacy despite the heterogeneity of test methods reflected by a high I^2^ measurement. Sensitivity analysis confirms that our sample size was robust as no single study drove efficacy outcomes, thus the quantity of data available were sufficient to overcome its heterogeneity. We also developed a framework for assessing risk of bias adapted from Cochrane methods, offering valuable insights for assessing this area of entomological research in a manner comparable to animal and epidemiological studies. Using this framework, most of our studies had low or unclear risk of bias, further supporting the strength of the evidence base collated in this study. Unclear risk of bias was most often reflected in the blinding category, where participants, investigators, and statisticians were often not blinded to treatment allocation or details on blinding were not reported. While mosquito count data is unlikely to be affected by blinding, future entomology studies can consider blinding investigators and statisticians, and using a placebo where possible as an extra caution to minimise the potential for bias. For study limitations, data sources were limited to academic studies although publication bias assessments did not suggest over-estimates of PE. Nine studies published between September 7, 2023 and July 28, 2025 were not included in the systematic review and meta-analysis but were summarized (Table 8). We do not expect these studies to substantially affect our systematic review and meta-analysis estimates, as results are generally consistent with our findings. The nine studies recently published reflect the rapidly growing evidence base for VPSRs, showing a promising trend with increased harmonisation of using HLC as an endpoint (used in 7/9 studies). While semi-field studies (2/9) showed high PE, field studies (7/9) showed varied results, suggesting that more field studies are needed to better understand these different impacts of VPSRs seen and how to use them. These studies can apply our ecological framework (Table 9) to understand VPSR use in different ecological conditions and use case scenarios.

In conclusion, VPSRs of any format offer protection from contact with mosquitoes. Test methodologies vary widely, showing that future harmonisation of testing and evaluation methodologies will help VPSR research. Methods development can draw on our insights that semi-field data reflects field data, and HLC provides the best quality measurements. Additional research priorities identified by gaps in data include a need for more field studies that evaluate outdoor protection in malaria-endemic settings, especially in West African, South American, and Southeast Asian settings. Addressing these research priorities is essential as this product class is further evaluated for public health use to prevent mosquito-borne diseases.

## Contributors

IC, SJM, and IEA conceptualised the study. IC, SJM, IEA, NFL, MFM, and FOO developed study methodology. IC conducted the literature search, IC, JKS, SLM, and DM screened literature, AM resolved discrepancies in literature search assessments. DM, SLM, IC, AGL, MO, and SG (Gowelo) extracted data for the systematic review. JKS, NA, MA, CSB, TC, JDC, GD, NE, UF, CF, SG (Gibson), NG, HK, DK, AL, NFL, MFM, AM, MM, ACM, WM, EPM, MN, SBO, FOO, WAO, JP, AP, AP, MS, FS, OS, JCS, CS, DS, MMT, JAT, EAV, GVP, JMW, CY, and SJM contributed original data for the meta-analysis; they are first or last authors on included studies in the meta-analysis, and the studies they contributed to are in the forest plot with the exception of eight publications that are detailed in a table on page 8 of the Appendix. SLM curated and analysed the data, the dataset and underlying data was accessed and verified by IC. IC, SLM, IEA, and SJM interpreted the data. IC and SJM wrote the first draft of the paper. SH provided insights on the manuscript. All authors read and approved the final version of the manuscript.

## Data sharing statement

While data from this study cannot be made available due to data sharing agreements, co-authors can be contacted for individual study-level data for studies included in the meta-analysis. Those who were first or last authors on studies included in the meta-analysis, comprised of all studies in the forest plot except for eight publications (detailed on page 8 of the Appendix), shared their data for this study. Some data were made available without additional data sharing agreements, while others had processes to agree on what the data would be used for. The corresponding author can be contacted for guidance regarding obtaining data from individual studies included in the meta-analysis. Source code is provided in the Appendix Section 5.

## Declaration of interests

JKS received research funds through the Ifakara Health Institute to conduct product evaluation on spatial repellents, NA received a Unitaid grant to the University of Notre Dame to conduct research on spatial repellents, CSB received payments to the University of Florida to fund mosquito research on spatial repellent development, NE received grants from the U.S. Department of Defense and U.S. Military Infectious Diseases Research Program to conduct research on spatial repellents, SH is an employee of Envu (2022 ES Deutschland GmbH) but does not have a conflict of interest with the study because he was not involved in data collection or analysis, NFL received a Unitaid grant on spatial repellents, and ACM is on an advisory board to the Peruvian Ministry of Health on dengue and vector control. SJM has service contracts for spatial repellent research from SC Johnson, Envu, Sumitomo, and Widder Bros. All other authors declare no conflicts of interest related to this study.
