# Supplementary material for: Volatile pyrethroid spatial repellents for preventing mosquito bites: a systematic review and meta-analysis
Source: eBioMedicine. 2025 Aug 26;119:105891. doi: 10.1016/j.ebiom.2025.105891 (PMC12789705; doi:10.1016/j.ebiom.2025.105891)
Supplement: Appendix [file mmc1.docx]

**Volatile pyrethroid spatial repellents for preventing mosquito bites:**

**a systematic review and meta-analysis**

Ingrid Chen, Sarah L Miller, Daniel Msellemu, Aidi Lugenge, Johnson Kyeba Swai, Nicole Achee, Marta Andrés, Christopher S. Bibbs, Theeraphap Chareonviriyaphap, J Derek Charlwood, Greg Devine, Noel Elman, Ulrike Fillinger, Carmen Flores-Mendoza, Seth Gibson, Nicodem Govella, Steven Gowelo, Sebastian Horstmann, Hitoshi Kawada, Daniel Kline, Aaron Lloyd, Neil F Lobo, Marta F Maia, Arnold Mmbando, Mara Moreno-Gómez, Amy Morrison, Winifrida Mponzi, Emmanuel P Mwanga, Margaret Njoroge, Sheila B Ogoma, Fredros O Okumu, Mercy Opiyo, Welbeck A Oumbouke, John Paliga, Arissara Pongsiri, Alongkot Ponlawat, Manop Saeaung, Ferdinand Salazar, Onyango Sangoro, Jennifer C Stevenson, Chutipong Sukkanon, Din Syafruddin, Mgeni Mohamed Tambwe, Julie-Anne A Tangena, Elodie A Vajda, Gonzalo Vazquez-Prokopec, Joseph M Wagman, Chanly Yan, Isabel Elaine Allen, Sarah J Moore

**Appendix**

**Contents**

1. Search concepts and strings (2-3)
2. Systematic review data and sources (4-8)
3. Meta-analysis variable coding (9)
4. Sensitivity analysis results (10-12)
5. Source code (13)
6. **Search concepts and strings**

There were two parts to the literature search. The first was identification of studies via databases and registers. The second was identification of studies via other methods (described in main text).

*Part 1. Identification of studies via databases and registers*

Concepts:

A table of concepts was generated as well as synonyms for those concepts to generate a search string for Pubmed. Further searches were then conducted using permutations of the search string targeting <150 results, as described below. Embase was then used to seek studies not found on Medline (Pubmed). Web of Science was then used searching cited references on a few searches done in Pubmed and Embase.

| **Concepts** | **Synonyms** | | | | | | | |
| --- | --- | --- | --- | --- | --- | --- | --- | --- |
| Volatile pyrethroid | Transfluthrin | Metofluthrin | Allethrin | Prallethrin | Meperfluthrin |  |  |  |
| Spatial repellent | Emanator | Push-pull | Coil | Eave ribbon |  |  |  |  |
| Mosquito | Vector control | Bite prevention | Anopheles | Aedes | Culex | Malaria | Dengue | Infectious disease |

PubMed

*Search strings*

- (Volatile pyrethroid OR transfluthrin OR metofluthrin OR allethrin OR prallethrin OR meperfluthrin) AND (spatial repellent OR emanator OR push-pull OR coil OR eave ribbon) AND (mosquito OR vector control OR bite prevention OR anopheles OR aedes OR culex OR malaria OR dengue OR infectious disease)
- (spatial repellent OR emanator OR push-pull OR coil OR eave ribbon) AND (mosquito OR vector control OR bite prevention OR anopheles OR aedes OR culex OR malaria OR dengue OR infectious disease)

Based on findings above, specialized search strings were also generated for the Hessian fabric prototype:

- (transfluthrin AND (spatial repellent OR emanator) AND (sisal OR eave ribbon OR hessian) AND (mosquito OR vector control OR bite prevention OR anopheles OR aedes OR culex OR malaria OR dengue OR infectious disease)
- transfluthrin AND (sisal OR eave ribbon OR hessian) AND (mosquito OR vector control OR bite prevention OR anopheles OR aedes OR culex OR malaria OR dengue OR infectious disease)

*Pubmed search dates:*

- First round of searches conducted August 24, 2021 and September 3, 2021
- Second round of searches conducted December 19, 2022
- Third round of searches conducted September 6, 2023

Embase

*Search strings*:

- (coil AND (mosquito OR vector control OR bite prevention OR anopheles OR aedes OR culex OR malaria OR dengue OR infectious disease)
- (push-pull AND mosquito)
- (spatial repellent AND mosquito)

*Embase search dates:*

- First round of searches conducted September 7 2021
- Second round of searches conducted December 19, 2022
- Third round of searches conducted September 6, 2023

Web of science

*Search strings:*

- (Volatile pyrethroid OR transfluthrin OR metofluthrin OR allethrin OR prallethrin OR meperfluthrin) AND (spatial repellent OR emanator OR push-pull OR coil OR eave ribbon) AND (mosquito OR vector control OR bite prevention OR anopheles OR aedes OR culex OR malaria OR dengue OR infectious disease)
- (spatial repellent AND mosquito)

*Web of Science search dates:*

- First round of searches conducted September 7 2021
- Second round of searches conducted December 19, 2022
- Third round of searches conducted September 6, 2023

The methods described above were applied to identify studies eligible for systematic review and analysis.

Additional studies published between September 7, 2023 and July 28, 2025 were identified using the same methods, conducted on July 28, 2025.

1. **Systematic review data and sources**

For the systematic review, the requirement for forest plot inclusion was the ability to run a Generalized Linear Mixed Model (GLMM) analysis. This was either through original data provided by study authors, or from the publication. Mosquito counts, protective efficacy, and the data source for the forest plot are shown in the table below. *Differences refers to reasons for separating one publication into more than one study.

| **Study** | | **Protective efficacy (%)** | **95% CI** | | **Mosquitoes** | | **Data source** |
| --- | --- | --- | --- | --- | --- | --- | --- |
| **Entry** | **Differences*** |  | **Low** | **High** | **Treatment** | **Control** |  |
| Lukwa (Zimbabwe) 2008 | metofluthrin | 0.92 | 0.81 | 1.00 | 80 / 1105 | 1025 / 1105 | Paper |
| Lukwa (Zimbabwe) 2008 | esbiothrin | 0.85 | 0.70 | 1.00 | 150 / 1175 | 1025 / 1105 | Paper |
| Tangena (Laos) 2018 |  | 0.83 | 0.73 | 0.90 | 1207 / 6439 | 5232 / 6439 | Dataset |
| Maia (Tanzania) 2016 |  | 0.80 | 0.78 | 0.82 | 721 / 4342 | 3621 / 4342 | Paper |
| Oumbouke (Benin) 2017 |  | 0.73 | 0.72 | 0.73 | 2795 / 8103 | 5308 / 8103 | Dataset |
| Achee (Thailand) 2012 |  | 0.45 | 0.14 | 0.76 | 20 / 68 | 48 / 68 | Paper |
| Ogoma (Tanzania) 2014 | transfluthrin | 0.38 | 0.13 | 0.53 | 3881 / 9531 | 5650 / 9531 | Paper |
| Syafruddin (Indonesia) 2014 |  | 0.33 | 0.05 | 0.58 | 939 /2345 | 1406 / 2345 | Paper |
| Ogoma (Tanzania) 2014 | metofluthrin | 0.30 | 0.02 | 0.50 | 4249 / 9899 | 5650 / 9899 | Paper |
| Salazar (Thailand) 2013 |  | -0.03 | -0.03 | -0.02 | 511 / 1025 | 514 / 1025 | Dataset |
| Vajda (Thailand) 2023 | human-baited net trap | 1.00 | 0.99 | 1.00 | 0 / 2080 | 2080 / 2080 | Paper |
| Vajda (Thailand) 2023 | human landing catch | 0.99 | 0.99 | 1.00 | 112 / 2704 | 2592 / 2704 | Paper |
| Zarella (United States) 2022 | outdoors | 0.90 | 0.61 | 0.99 | 84 / 930 | 846 / 930 | Paper |
| Kawada (Indonesia) 2005 |  | 0.88 | 0.76 | 0.94 | 1481 / 5867 | 4386 / 5867 | Dataset |
| McPhatter (United States of America) 2017 |  | 0.75 | 0.42 | 0.90 | 223 / 1120 | 897 / 1120 | Dataset |
| Zarella (United States) 2022 | indoors | 0.75 | 0.42 | 0.98 | 104 / 436 | 332 / 436 | Paper |
| Swai (Tanzania) 2023 |  | 0.70 | 0.57 | 0.78 | 5591 / 21983 | 16392 / 21983 | Paper |
| Stevenson (Zambia) 2018 | indoors | 0.64 | 0.60 | 0.67 | 1329 / 3522 | 2193 / 3522 | Paper |
| Kawada (Tanzania) 2008 | spray collection | 0.55 | 0.41 | 0.66 | 505 / 1476 | 971 / 1476 | Dataset |
| Devine (Mexico) 2021 |  | 0.50 | 0.35 | 0.62 | 5299 / 17027 | 11728 / 17027 | Dataset |
| Charlwood (Cambodia) 2014 |  | 0.47 | -0.10 | 0.74 | 4259 / 22773 | 18514 / 22773 | Dataset |
| Charlwood (Cambodia) 2016 |  | 0.39 | 0.16 | 0.56 | 4038 / 8803 | 4765 / 8803 | Paper |
| Burton (Zambia) 2023 |  | 0.39 | 0.35 | 0.43 | 4319 / 11352 | 7033/ 11352 | Paper |
| Charlwood (Cambodia) 2017 |  | 0.37 | -0.04 | 0.62 | 351 / 1851 | 1500 / 1851 | Dataset |
| Flores-Mendoza (Peru) 2022 |  | 0.35 | 0.19 | 0.47 | 2473 / 6162 | 3689 / 6162 | Paper |
| Morrison (Peru) 2022 |  | 0.26 | 0.15 | 0.37 | 160459 / 368017 | 207558 / 368017 | Paper |
| Syafruddin (Indonesia) 2020 | indoors | 0.23 | -0.02 | 0.41 | 3883 / 8764 | 4881 / 8764 | Dataset |
| Permana (Indonesia) 2022 | indoors | 0.17 | 0.04 | 0.28 | 6770 / 14559 | 7789 / 14559 | Dataset |
| Permana (Indonesia) 2022 | outdoors | 0.15 | -0.05 | 0.31 | 4372 / 9206 | 4834 / 9206 | Dataset |
| Syafruddin (Indonesia) 2020 | outdoors | 0.12 | -0.17 | 0.34 | 4372 / 9194 | 4822 / 9194 | Dataset |
| Kawada (Tanzania) 2008 | light trap | 0.02 | -0.32 | 0.28 | 2179 / 4321 | 2142 / 4321 | Dataset |
| Stevenson (Zambia) 2018 | outdoors | -0.01 | -0.13 | 0.11 | 2967 / 4976 | 2009 / 4976 | Paper |
| Lloyd (United States of America) 2013 | allethrin | 0.69 | 0.45 | 0.82 | 76 / 334 | 258 / 334 | Dataset |
| Lloyd (United States of America) 2013 | metofluthrin | 0.63 | 0.37 | 0.78 | 97 / 355 | 258 / 355 | Dataset |
| Britch (United States of America) 2021 | metofluthrin | 0.60 | 0.14 | 0.81 | 20185 / 59762 | 39577 / 59762 | Dataset |
| Frances (Australia) 2020 |  | 0.52 | 0.42 | 0.61 | Not available | Not available | Paper |
| Britch (United States of America) 2021 | allethrin | 0.48 | -0.67 | 0.84 | 25793 / 51742 | 25949 / 51742 | Dataset |
| Dame (United States of America) 2014 | allethrin | 0.41 | 0.23 | 0.55 | 4317 / 11636 | 7319 / 11636 | Dataset |
| Dame (United States of America) 2014 | metofluthrin | 0.04 | -0.34 | 0.31 | 6975 / 11636 | 7319 / 11636 | Dataset |
| Mmbando (Tanzania) 2018 | outdoor, semi-field | 0.99 | 0.99 | 1.00 | 2068 / 8619 | 6551 / 8619 | Dataset |
| Ogoma (Tanzania) 2012 |  | 0.98 | 0.97 | 0.99 | 22 / 916 | 894 / 916 | Dataset |
| Mmbando (Tanzania) 2018 | indoor, semi-field | 0.95 | 0.90 | 0.98 | 152 / 826 | 674 / 826 | Dataset |
| Govella (Tanzania) 2015 |  | 0.95 | 0.92 | 0.97 | 1479 /20240 | 18761 / 20240 | Dataset |
| Njoroge (Kenya) 2022 | indoors | 0.92 | 0.84 | 0.96 | 205 / 2560 | 2355 / 2560 | Paper |
| Tambwe (Tanzania) 2021 | human landing catch | 0.88 | 0.85 | 0.91 | 192 / 760 | 568 / 760 | Paper |
| Masalu (Tanzania) 2018 | human landing catch | 0.88 | 0.83 | 0.91 | 440 / 2184 | 1744 / 2184 | Dataset |
| Mmbando (Tanzania) 2017 | indoors | 0.86 | 0.72 | 0.93 | 55 / 443 | 388 / 443 | Paper |
| Masalu (Tanzania) 2017 |  | 0.84 | 0.77 | 0.88 | 1681 / 10579 | 8898 / 10579 | Dataset |
| Njoroge (Kenya) 2021 |  | 0.83 | 0.77 | 0.88 | 1703 / 4904 | 3201 / 4904 | Paper |
| Andrés (Tanzania) 2015 |  | 0.83 | 0.71 | 0.91 | 339 / 1430 | 1091 / 1430 | Paper |
| Njoroge (Kenya) 2022 | outdoors | 0.78 | 0.62 | 0.88 | 473 / 2560 | 2087 / 2560 | Paper |
| Ogoma (Tanzania) 2017 | human landing catch | 0.75 | 0.67 | 0.81 | 29960 / 220443 | 190483 / 220443 | Dataset |
| Yan (Thailand) 2023 | jute | 0.73 | 0.26 | 0.90 | 145 / 674 | 529 / 674 | Paper |
| Swai (Tanzania) 2019 | indoors | 0.70 | 0.67 | 0.73 | 5186 / 22031 | 16845 / 22031 | Paper |
| Masalu (Tanzania) 2018 | trap density | 0.69 | 0.51 | 0.80 | 3614 / 9776 | 6162 / 9776 | Paper |
| Sukkanon (Thailand) 2021 | semi-field | 0.66 | 0.56 | 0.75 | 65 / 262 | 197 / 262 | Paper |
| Sangoro (Tanzania) 2020 | field | 0.66 | 0.59 | 0.71 | 2030 / 7028 | 4998 / 7028 | Dataset |
| Mmbando (Tanzania) 2017 | outdoors | 0.64 | 0.41 | 0.77 | 921 / 3127 | 2206 / 3127 | Dataset |
| Sangoro (Tanzania) 2020 | semi-field | 0.61 | 0.48 | 0.71 | 11223 / 28713 | 17490 / 28713 | Dataset |
| Tambwe (Tanzania) 2020 |  | 0.61 | 0.52 | 0.70 | 439 / 1365 | 926 / 1365 | Paper |
| Tambwe (Tanzania) 2021 | trap density | 0.61 | 0.56 | 0.65 | 336 / 1200 | 864 / 1200 | Paper |
| Tambwe (Tanzania) 2021 |  | 0.60 | 0.55 | 0.63 | 1765 / 6382 | 4617 / 6382 | Dataset |
| Fillinger (Kenya) 2023 | trap density | 0.59 | 0.51 | 0.65 | 4049 / 11599 | 7550 / 11599 | Dataset |
| Wagman (Belize) 2015 |  | 0.51 | 0.38 | 0.62 | 1646 / 5308 | 3662 / 5308 | Dataset |
| Masalu (Tanzania) 2020 |  | 0.52 | 0.47 | 0.56 | 10610 / 32738 | 22128 / 32738 | Dataset |
| Mponzi (Tanzania) 2022 |  | 0.50 | 0.42 | 0.57 | 5214 / 15724 | 10510 / 15724 | Dataset |
| Mmbando (Tanzania) 2023 | outdoors | 0.46 | 0.32 | 0.57 | 763 / 2188 | 1425 / 2188 | Dataset |
| Swai (Tanzania) 2019 | outdoors | 0.46 | 0.23 | 0.62 | 283 / 829 | 546 / 829 | Dataset |
| Mmbando (Tanzania) 2018 | outdoors | 0.45 | 0.28 | 0.58 | 1686 / 4918 | 3232 / 4918 | Dataset |
| Tambwe (Tanzania) 2023 |  | 0.44 | 0.23 | 0.59 | 1872 / 3890 | 2018 / 3890 | Paper |
| Britch (United States) 2020 | indoors | 0.43 | 0.31 | 0.64 | 110 / 282 | 172 / 282 | Dataset |
| Mmbando (Tanzania) 2018 | indoors | 0.41 | 0.26 | 0.53 | 4558 / 12361 | 7803 / 12361 | Dataset |
| Mmbando (Tanzania) 2023 | indoors | 0.40 | 0.19 | 0.55 | 30322 / 67273 | 36951 / 67273 | Dataset |
| Fillinger (Kenya) 2023 | human landing catch | 0.34 | 0.26 | 0.41 | 9416 / 22805 | 13389 / 22805 | Dataset |
| Yan (Thailand) 2023 | cotton | 0.33 | -0.82 | 0.76 | 352 / 856 | 504 / 856 | Paper |
| Tangena (Laos) 2018 |  | 0.31 | 0.18 | 0.42 | 1924 / 4679 | 2755 / 4679 | Paper |
| Salazar (Thailand) 2013 |  | 0.25 | 0.10 | 0.37 | 2383 / 5989 | 3606 / 5989 | Dataset |
| Sukkanon (Thailand) 2021 | field | 0.21 | 0.16 | 0.24 | 1429 / 3227 | 1798 / 3227 | Paper |
| Ogoma (Tanzania) 2017 | trap density | 0.18 | 0.10 | 0.25 | 25451 / 99418 | 73967 / 99418 | Dataset |
| Britch (United States) 2020 | outdoors | 0.16 | -0.08 | 0.34 | 465 / 1103 | 638 / 1103 | Dataset |
| Moreno-Gómez (Italy) 2021 |  | 0.73 | 0.63 | 0.80 | 5163 / 15311 | 10148 / 15311 | Dataset |
| Bibbs (United States) 2021 | mixed pyrethoids | 0.49 | 0.23 | 0.67 | 2680 / 6588 | 3908 / 6588 | Dataset |
| Bibbs (United States) 2021 | sumithrin | 0.45 | -0.37 | 0.78 | 465 / 1442 | 977 / 1442 | Dataset |
| Bibbs (United States) 2021 | permethrin | 0.36 | -0.14 | 0.64 | 707 / 1684 | 977 / 1684 | Dataset |
| Bibbs (United States) 2021 | metofluthrin | -0.01 | -2.22 | 0.68 | 1504 / 2481 | 977 / 2481 | Dataset |

While most references found clearly met or did not meet inclusion criteria, the following references were not included in the forest plot because they were before-after studies, and therefore did not account for temporal heterogeneity that can be a source of bias.

| 1 | Xue, R. D., Qualls, W. A., Smith, M. L., Gaines, M. K., Weaver, J. H., & Debboun, M. (2012). Field evaluation of the Off! Clip-on Mosquito Repellent (metofluthrin) against Aedes albopictus and Aedes taeniorhynchus (Diptera: Culicidae) in northeastern Florida. Journal of medical entomology, 49(3), 652–655. https://doi.org/10.1603/me10227 |
| --- | --- |
| 2 | Buhagiar, T. S., Devine, G. J., & Ritchie, S. A. (2017). Metofluthrin: investigations into the use of a volatile spatial pyrethroid in a global spread of dengue, chikungunya and Zika viruses. Parasites & vectors, 10(1), 270. https://doi.org/10.1186/s13071-017-2219-0 |
| 3 | Ritchie, S. A., & Devine, G. J. (2013). Confusion, knock-down and kill of Aedes aegypti using metofluthrin in domestic settings: a powerful tool to prevent dengue transmission?. Parasites & vectors, 6(1), 262. <https://doi.org/10.1186/1756-3305-6-262> |

The following references were not included in the forest plot because protective efficacy estimates could not be extracted. The references are otherwise included in the systematic review.

| 1 | Pates, H. V., Line, J. D., Keto, A. J., & Miller, J. E. (2002). Personal protection against mosquitoes in Dar es Salaam, Tanzania, by using a kerosene oil lamp to vaporize transfluthrin. Medical and veterinary entomology, 16(3), 277–284. https://doi.org/10.1046/j.1365-2915.2002.00375.x |
| --- | --- |
| 2 | Msangi, S., Mwang'onde, B. J., Mahande, A. M., & Kweka, E. J. (2010). Field Evaluation of the Bio-Efficacy of Three Pyrethroid Based Coils against Wild Populations of Anthropophilic Mosquitoes in Northern Tanzania. Journal of global infectious diseases, 2(2), 116–120. https://doi.org/10.4103/0974-777X.62885 |
| 3 | Kitau, J., Pates, H., Rwegoshora, T. R., Rwegoshora, D., Matowo, J., Kweka, E. J., Mosha, F. W., McKenzie, K., & Magesa, S. M. (2010). The effect of Mosquito Magnet Liberty Plus trap on the human mosquito biting rate under semi-field conditions. Journal of the American Mosquito Control Association, 26(3), 287–294. https://doi.org/10.2987/09-5979.1 |
| 4 | Rapley, L. P., Russell, R. C., Montgomery, B. L., & Ritchie, S. A. (2009). The effects of sustained release metofluthrin on the biting, movement, and mortality of Aedes aegypti in a domestic setting. The American journal of tropical medicine and hygiene, 81(1), 94–99. |
| 5 | Revay, E. E., Junnila, A., Xue, R. D., Kline, D. L., Bernier, U. R., Kravchenko, V. D., Qualls, W. A., Ghattas, N., & Müller, G. C. (2013). Evaluation of commercial products for personal protection against mosquitoes. Acta tropica, 125(2), 226–230. https://doi.org/10.1016/j.actatropica.2012.10.009 |
| 6 | Xue, R. D., Qualls, W. A., Smith, M. L., Gaines, M. K., Weaver, J. H., & Debboun, M. (2012). Field evaluation of the Off! Clip-on Mosquito Repellent (metofluthrin) against Aedes albopictus and Aedes taeniorhynchus (Diptera: Culicidae) in northeastern Florida. Journal of medical entomology, 49(3), 652–655. https://doi.org/10.1603/me10227 |

The following references were not included in the individual mosquito-level data meta-analysis because data could not be made available.

|  | **Citation** | **Reason** |
| --- | --- | --- |
| 1 | Frances, S. P., Rowcliffe, K. L., & MacKenzie, D. O. (2020). Field Effectiveness of a Metofluthrin Fan-Based Emanator and Deet as Repellents Against Aedes vigilax in Southeast Queensland, Australia. Journal of the American Mosquito Control Association, 36(2), 120–122. https://doi.org/10.2987/19-6905.1 | No response from authors |
| 2 | Rapley, L. P., Russell, R. C., Montgomery, B. L., & Ritchie, S. A. (2009). The effects of sustained release metofluthrin on the biting, movement, and mortality of Aedes aegypti in a domestic setting. The American journal of tropical medicine and hygiene, 81(1), 94–99. | No response from authors |
| 3 | Revay, E. E., Junnila, A., Xue, R. D., Kline, D. L., Bernier, U. R., Kravchenko, V. D., Qualls, W. A., Ghattas, N., & Müller, G. C. (2013). Evaluation of commercial products for personal protection against mosquitoes. Acta tropica, 125(2), 226–230. https://doi.org/10.1016/j.actatropica.2012.10.009 | Data could not be located |
| 4 | Achee, N., Masuoka, P., Smith, P., Martin, N., Chareonviryiphap, T., Polsomboon, S., Hendarto, J., & Grieco, J. (2012). Identifying the effective concentration for spatial repellency of the dengue vector Aedes aegypti. Parasites & vectors, 5, 300. https://doi.org/10.1186/1756-3305-5-300 | No response from authors |
| 5 | Pates, H. V., Line, J. D., Keto, A. J., & Miller, J. E. (2002). Personal protection against mosquitoes in Dar es Salaam, Tanzania, by using a kerosene oil lamp to vaporize transfluthrin. Medical and veterinary entomology, 16(3), 277–284. https://doi.org/10.1046/j.1365-2915.2002.00375.x | Data could not be located |
| 6 | Kitau, J., Pates, H., Rwegoshora, T. R., Rwegoshora, D., Matowo, J., Kweka, E. J., Mosha, F. W., McKenzie, K., & Magesa, S. M. (2010). The effect of Mosquito Magnet Liberty Plus trap on the human mosquito biting rate under semi-field conditions. Journal of the American Mosquito Control Association, 26(3), 287–294. https://doi.org/10.2987/09-5979.1 | Data could not be located |
| 7 | Msangi, S., Mwang'onde, B. J., Mahande, A. M., & Kweka, E. J. (2010). Field Evaluation of the Bio-Efficacy of Three Pyrethroid Based Coils against Wild Populations of Anthropophilic Mosquitoes in Northern Tanzania. Journal of global infectious diseases, 2(2), 116–120. https://doi.org/10.4103/0974-777X.62885 | Data could not be located |
| 8 | Lukwa, N., & Chiwade, T. (2008). Lack of insecticidal effect of mosquito coils containing either metofluthrin or esbiothrin on Anopheles gambiae sensu lato mosquitoes. Tropical biomedicine, 25(3), 191–195. | No response from authors |

Changes to study protocol registration on PROSPERO CRD42021268852: planned approach for data synthesis did not stratify by timepoint (1-14 days, 2-4 weeks, 1-3 months, 3-6 months, ≥6 months) because most studies were under one month in duration. We also presented the meta-analysis aggregate data by subgroup in a pooled forest plot, as the originally proposed plot of protective efficacy vs. time elapsed since initial treatment was not applicable for reasons stated above. The survival analysis was therefore also not applicable to this study. For various secondary entomological outcomes, this was summarized descriptively due to limited data available as well. Subgroup analysis on outdoor weather was not conducted as results were not sensitive to weather.

1. **Meta-analysis variable coding**

The table below shows the variables extracted for the pooled meta-analysis.

| **Variable** | **Details** |
| --- | --- |
| *Study descriptors* | |
| Author and year of publication | (string) |
| Country | (string) |
| Intervention format | passive fabric, commercial passive, commercial energy, coil, spray |
| Active ingredient | transfluthrin, sumithrin, metofluthrin, allethrin, permethrin, mixed |
| Capture method | human landing catch, trap density |
| Trap type | human-baited, odor-baited mechanical, light, floor, exit |
| Setting* | indoors, outdoors |
| Study type* | Field, semi-field |
| *Treatment / control* | |
| Treatment allocation | treatment = 1  control = 0 |
| Mosquito counts | treatment, control, total |
| Mosquito species | aedes (total, aegypti, albopictus, other)  anopheles (total, arabiensis, gambiae, other)  culex total |
| *Additional variables* | |
| Experimental day | (dates were converted to experimental days when necessary) |
| Hut/chamber id | (hut or indoor studies only: each unique hut or chamber was given a unique number) |
| Location id | (field studies: each unique location/village was given a unique number) |
| Volunteer id | (each unique volunteer who collected mosquitoes was given a unique number) |
| Temperature | average temperature reported in study |
| Humidity | average humidity reported in study |
| Terraclimate temperature | temperature reported based on geo coordinates over the study period (included both max, min, and average temp) |
| Terraclimate vapor pressure | vapor pressure reported based on geo coordinates over the study period |
| Terraclimate wind | wind reported based on geo coordinates over the study period |

*Semifield indoors = huts in tented enclosures, all other semifield studies were categorized as outdoor.

1. **Sensitivity analysis results**

Sensitivity analysis was conducted using terraclimate weather variables: temperature (max, min, average), humidity, wind, and combined weather effects. The effect of removing one study at a time on overall protective efficacy was also investigated. Results are shown below.

Climate analysis

|  | **Protective Efficacy (1 – IRR)** | | **95% Confidence Interval** | | | **p-value** |
| --- | --- | --- | --- | --- | --- | --- |
| **Variables** |  |  | | |  | |
| Unadjusted model | 0.52 | 0.42 – 0.60 | | <0.001 | | |
| Maximum temperature | 0.51 | 0.41 – 0.60 | | | <0.001 | |
| Minimum temperature | 0.51 | 0.40 – 0.60 | | | <0.001 | |
| Average temperature | 0.51 | 0.40 – 0.59 | | | <0.001 | |
| Average vapor pressure | 0.51 | 0.39 – 0.61 | | | <0.001 | |
| Average wind speed | 0.52 | 0.44 – 0.59 | | | <0.001 | |
| Average temperature, average vapor pressure, average wind speed | 0.51 | 0.41 – 0.59 | | | <0.001 | |
| The unadjusted model is a random effects model using a negative binomial distribution and includes the following variables: mosquito repellent treatment as the exposure, mosquito landing or trap counts as the outcome, and day as a random effect. Climate variables are derived from terraclimate information and contain monthly averages. | | | | | | |

In the unadjusted model, protective efficacy was estimated to be 52% (95% CI 42% - 60%). No difference in protective efficacy was observed when we adjusted for wind speed. When we adjusted for maximum temperature alone, minimum temperature alone, average temperature alone, average vapor pressure alone, or average temperature, average vapor pressure, and average wind speed together, VPSR protective efficacy was estimated to be 51%, with 95% CI falling between 39-61%. These results suggest the included climate variables had little impact on VPSR protective efficacy.

**Below is the model output when average temperature, vapor pressure, and wind speed are adjusted for.**

|  | **IRR (95% Confidence Interval)** | **Protective Efficacy**  **(1 – IRR)** | **95% Confidence Interval** | | **p-value** |
| --- | --- | --- | --- | --- | --- |
| **Variables** |  |  |  |  | |
| Unadjusted model treatment effect | 0.48 (0.40 – 0.58) | 0.52 | 0.42 – 0.60 | <0.001 | |
| Treatment | 0.49 (0.41 – 0.59) | 0.51 | 0.41 – 0.59 | <0.001 | |
| Average temperature | 0.95 (0.89 – 1.02) | 0.05 | -0.02 – 0.11 | 0.138 | |
| Average vapor pressure | 0.57 (0.28 – 1.18) | 0.43 | -0.18 – 0.72 | 0.133 | |
| Average wind speed | 0.70 (0.62 – 0.78) | 0.30 | 0.22 – 0.38 | <0.001 | |

In the unadjusted model, protective efficacy was estimated to be 52% (95% CI 42% - 60%). When adjusting for average temperature, average vapor pressure, and average wind speed, VPSR protective efficacy was estimated to be 51% (95% CI 41% - 59%). Generally, we found that protective efficacy was positively correlated with increasing temperature, vapor pressure, and wind speed. Average wind speed was statistically significant, however, the treatment effect was largely unchanged when adjusted for wind speed. Still, future studies may benefit from measuring climate variables in order to better understand VPSR efficacy in different settings.

Comparison of terraclimate data and weather station data

**
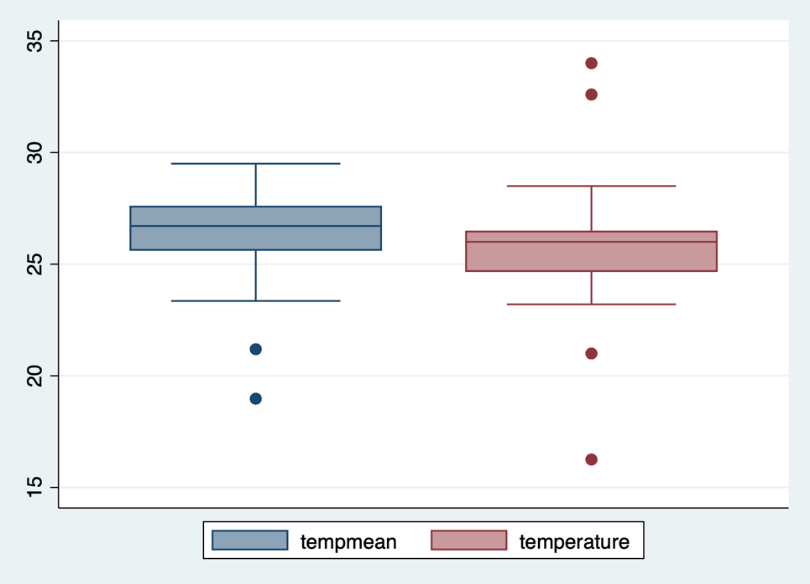
**

Temp mean = terraclimate (26.51 °C)

Temperature = raw data (25.02 °C)


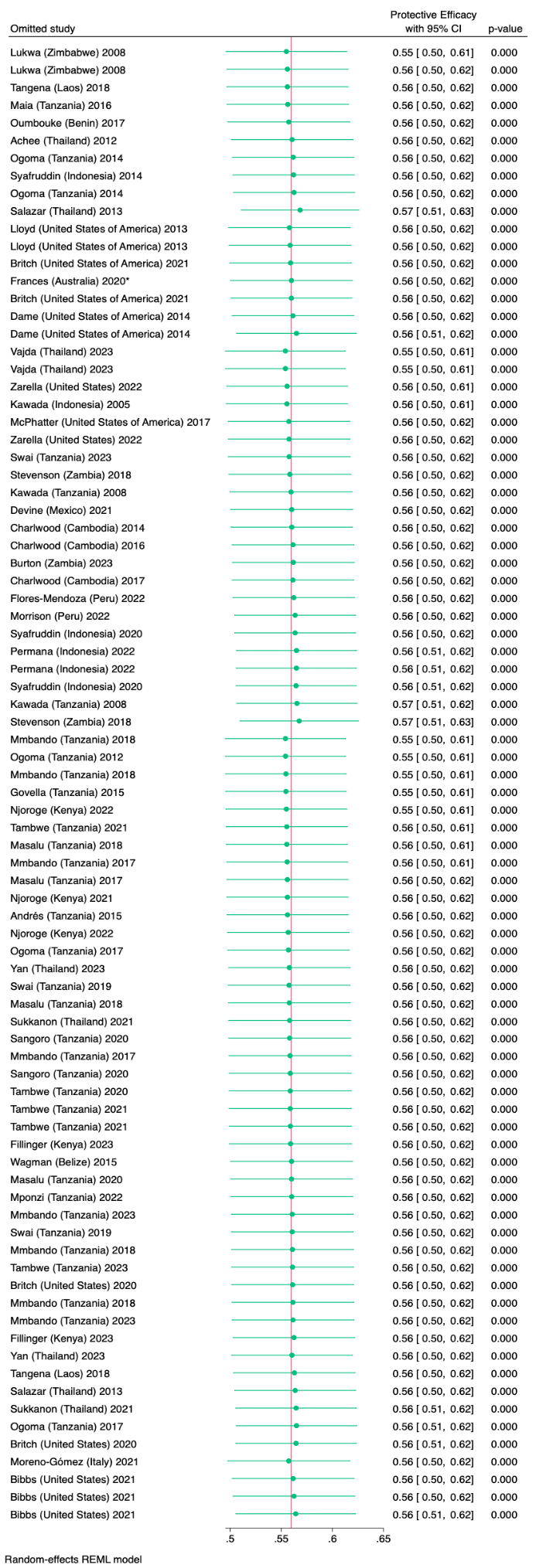
Leave one out analysis (*p*-value 0.000 means <0.0005)

1. **Source code**

Code for Stata 18.5

1. Negative binomial mixed effects model:

menbreg totalcount treatment ||day:, irr vce(robust)

where totalcount = mosquito count outcome and treatment = VPSR treatment

1. Meta-analysis/forest plot for study-level:

meta set pe pe_ll pe_ul, civartolerance(1e0) studylabel(authyr) studysize(size) eslabel(Protective Efficacy)

meta forestplot _id treatment control _plot _esci _weight comment id, random(reml) subgroup(IF) esrefline nullrefline

1. Meta-analysis/forest plot for pooled:

meta set pe pe_ll pe_ul, civartolerance(1e0) studylabel(label) eslabel(Protective Efficacy)

meta forestplot _id treatment control _plot _weight _esci, random(reml) subgroup(category) esrefline nullreflin
