# Supplementary material for: Volatile pyrethroid spatial repellents for preventing mosquito bites: a systematic review and meta-analysis
Source: eBioMedicine. 2025 Aug 26;119:105891. doi: 10.1016/j.ebiom.2025.105891 (PMC12789705; doi:10.1016/j.ebiom.2025.105891)
Supplement: Supplemental Tables [file mmc2.docx]

**Table S1. Mosquito experimental conditions and entomological outcomes**

| **Author & year** | **If semi-field, size release chamber (m)** | **Intervention Format** | **Mosquito lab conditions** | | | **Number of treatment days / nights** | **Entomological outcomes measured** | | **Ref** |
| --- | --- | --- | --- | --- | --- | --- | --- | --- | --- |
|  |  |  | **Strain** | **Age** | **Starvation Period** |  | **Knockdown** | **Mortality** |  |
| Lukwa (2008) | - | Coil | Strain not specified  3 to 5 day old female  Starved for 4 hrs | | | 1 day  1 night | Mean knockdown rate was 90% for metofluthrin | Metofluthrin formulations could not be classified as having insecticidal effect since did not meet the 95% mortality rate criteria | (1) |
| Lukwa (2008) | - | Coil | Strain not specified  3 to 5 day old female  Starved for 4 hrs | | | 1 day  1 night | Mean knockdown rate 73.3% for esbiothrin | Esbiothrin formulations could not be classified as having insecticidal effect since did not meet the 95% mortality rate criteria | (1) |
| Tangena (2018) | - | Coil | Wild | | | 14 days | Metofluthrin | 100% mortality was noted for permethrin (n= 148) | (2) |
| Maia (2016) | - | Coil | Wild with unknown age and period of starvation | | | 4 nights | No info | No info | (3) |
| Oumbouke (2017) | - | Coil | No info | | | 52 nights | No info | Induced mortality: percentage of dead mosquitoes in treated hut relative to percentage dead in control hut | (4) |
| Achee (2012) | - | Coil | Lab-reared  4 to 7 days  Starved for 24 hrs | | | 3 days | Minimal knockdown was observed in sentinel mosquito cohorts positioned adjacent. | A total of 8% (5/60) mortality was observed from sentinel cohorts inside the control hut (data not shown). | (5) |
| Ogoma (2014) | - | Coil | Wild *An. arabiensis*  in field  3 to 8 days old, sugar starved for 6 hrs | | | 64 nights | Collection was done at 0700 hours inside the huts to capture resting, knocked down mosquitoes | After 24 hours in field experiments was very low. Only 0.02% mortality of all mosquito species collected was observed. | (6) |
| Syafruddin (2014) | - | Coil | Wild strain | | | 4 nights | No info | No info | (7) |
| Ogoma (2014) | Ifakara design experimental huts | Coil | Reared *An. gambiae sensu stricto (s.s.)* | | | 64 nights | Collection was done at 0700 hours inside the huts to capture resting, knocked down mosquitoes | More than 90% of the mosquitoes collected in- side huts that had mosquito coils and DDT had died within 24 hours unlike in the control hut | (6) |
| Salazar (2013) | 4 × 3.5 × 40 | Coil | Lab-reared 3 to 5 days old  Sugar starved | | | No info | No significant differences in recapture rates among metofluthrin-exposed and both positive (blank coil) and negative control | No info | (8) |
| Vajda (2023) | - | Commercial passive | Lab-reared  female nulliparous 5 to 8 days old Sugar starved for 8 hrs | | | 32 nights | No info | No info | (9) |
| Zarella (2022) |  | Commercial passive | Lab-reared 3 to 7 days old  Starved for 24 hrs | | | 4 days | Knock-down assessed by counting mosquitoes exhibiting unusual behavior of being “knocked out” of the air due to paralysis from exposure | Mortality of mosquitoes exposed to metofluthrin emaNo infotors was ~2x higher than those who were not exposed in indoor conditions. | (10) |
| Kawada (2005) |  | Commercial passive | No info | | | 6 days and later 4 months | No info | No info | (11) |
| McPhatter (2017) | 6.1 x 10.7 x 3.4 | Commercial passive | Lab-reared  5 to 10 days old  Starved for 24 hrs | | | No info | In this study, knockdown effects from transfluthrin expo­sure were low | No info on mortality but reduction of entry into a tent structure | (12) |
| Zarella (2022) |  | Commercial passive | Lab-reared 3 to 7 days old  Starved for 24 hrs | | | 4days | Knock-down assessed by counting mosquitoes exhibiting unusual behavior of being “knocked out” of the air due to paralysis from exposure | Mortality of mosquitoes exposed to metofluthrin emanators was ~2x higher than those who were not exposed in indoor conditions. | (10) |
| Swai (2023) | 3.25 x 3.5 x 2 | Commercial passive | Wild strain | | | 32 nights | KD was 40% compared to 21% of control | Mosquito mortality at 24hrs was 52% significantly higher than the control huts 35% | (13) |
| Stevenson (2018) | 28.8 x 21 x 3 | Commercial passive | Lab-reared,  2 to 5 days old  Sugar starved for 6 hrs | | | 10 nights | No info | Mortality may be explained by excito-repellency activity of metofluthrin | (14) |
| Kawada (2008) | - | Commercial passive | Wild strain | | | 124days | No info | No info | (15) |
| Devine (2021) | - | Commercial passive | Lab-reared | | | 21 weeks | No info on KD | No info on mortality but reduction in vector densities and human-vector contact indoor | (16) |
| Charlwood (2014) | - | Commercial passive | Wild population | | | - | No info | No info | (17) |
| Charlwood (2016) | - | Commercial passive | Wild population | | | 16 nights | No info on KD but reduced landing | No info on mortality | (18) |
| Burton (2023) | 10 x10 x 3 | Commercial passive | Lab-reared  2 to 5 days old Sugar starved | | | 32 nights | Mosquito does not exhibit host-seeking behavior overnight, recovered dead in the morning | Direct mosquito mortality was calculated as the proportion of released mosquitoes found knocked down or dead. | (19) |
| Charlwood (2017) |  | Commercial passive | No info | | | 6 weeks | No info on KD | No info | (20) |
| Flores-Mendoza (2022) | - | Commercial passive | Wild strain | | | 7days | No info on KD | No info | (21) |
| Morrison (2022) | - | Commercial passive | Wild strain | | | Not applicable | Not applicable | Not applicable | (22) |
| Syafruddin (2020) | - | Commercial passive | Wild strain | | | 2 weeks | No info | No info | (23) |
| Permana (2022) | - | Commercial passive | Wild strain | | | 48 nights | No info | No info | (24) |
| Lloyd (2013) | - | Commercial Requiring Energy |  | | | No info | No info | No info | (25) |
| Britch (2021) | - | Commercial Requiring Energy | Lab-reared | | | 12 nights | Metofluthrin creates disorientation, allethrin is strong enough to repel at close range but does not accumulate sufficiently to disorient | No mortality information collected | (26) |
| Frances (2020) |  | Commercial Requiring Energy | No info | | | 3 nights | No info | On emanator caused mortality in mosquitoes placed 0.3 m away from it. However, the emanator was not effective when 5–8 km/h of airflow occurred. | (27) |
| Dame (2014) | - | Commercial Requiring Energy | No info | | | 12 nights | No info | No info | (28) |
| Mmbando (2018) | - | Passive fabric | Wild strain | | | 16 nights | No info | No info | (29) |
| Ogoma (2012) | 60 x 2 x 2.5 | Passive fabric | Lab-reared  Nulliparous female,  2 to 6 day old mosquitoes | | | 4 nights | No info | No info | (30) |
| Mmbando (2018) | 9.6 x 2.1 | Passive fabric | Lab reared  4 to 8 day old nulliparous female  Starved for 12 hrs | | | 75 (15 per treatment) | No info | Mortality was evaluated at each morning when retrieving the traps. Observation was made after 24 h. | (29) |
| Govella (2015) | - | Passive fabric | Lab-reared | | | 72 nights | No info | No info | (31) |
| Njoroge (2022) | 27 x 11 x 4.3 | Passive fabric | Lab-reared 3 to 5 days old  Starved for 5 hrs | | | 16 nights | No info | Twenty-four hours after  the 1-h exposure, a mortality of 93.2% was found, which  was corrected to 91.15% according to WHO guidelines. | (32) |
| Tambwe (2021) | 9 x 21 | Passive fabric | Lab-reared  3 to 8 days old  Starved for 12 hrs | | | 8 days | No info | No info | (33) |
| Masalu (2018) | - | Passive fabric | - | | | 27 nights |  | After 24 h, mosquitoes were sorted and recorded as dead, live, | (34) |
| Mmbando (2017) | - | Passive Fabric | Wild strain | | | No info | No info | No info | (35) |
| Masalu (2017) | - | Passive fabric | - | | | 3 nights | No info | No info | (36) |
| Njoroge (2021) | 11 x 7 x 2.5 | Passive fabric | Lab-reared 3 to 5 days old  Starved for 3 to 5 hrs | | | 16 days | No info | No info | (37) |
| Andrës (2015) | 9 x 28.8 | Passive fabric | Lab-reared  3 to 8 days old nulliparous Starved for 6 hrs | | | 32 nights | Impregnated strips continued to knock- down mosquitoes up to 3 weeks after | Data for 24-h mortality showed old strips caused 91% mortality compared to 28% mortality for 3-week old strips | (38) |
| Ogoma (2017) | - | Passive fabric | Wild | | | 12 nights | No info | No info | (39) |
| Yan (2023) | 40 x 3.5 x 4 | Passive fabric | Lab-reared  3 to 5 day old females  Non-blood fed  Sugar starved for 6 h | | | 16 nights | Knockdown reached a peak at 70.75% and of transfluthrin 20% and significantly decreased with lower con- centration | mortality reached a peak of 38.5%, at transfluthrin 20% and significantly decreased with lower concentration | (40) |
| Swai (2019) | - | Passive fabric | Wild | | | 16 nights | No info | No info | (41) |
| Sukkanon (2021) | 40 x 3.5 x 4 | Passive fabric | Wild strain | | | 2 days | No mosquito knockdown was observed each hour on the floor sheeting | No mortality was found in the control group of marked mosquitoes after the 24-h holding period | (42) |
| Sangoro (2020) | 36 ground x 4.5  110 x 2 x 2.5 | Passive fabric | Lab-reared 4 to 9 days old | | | 12 nights | No info | No info | (43) |
| Tambwe (2020) | 9 x 21 | Passive fabric | Lab reared  3 to 8 day old female *Aedes*  sugar-starved for 12 h | | | 16 nights | No info | No info | (44) |
| Tambwe (2021) | 21 x 29 x 4.5 | Passive fabric | Lab-reared 3 to 8 day old Starved for 6 hrs | | | 16 days | No info | No significant difference in 24h mortality was observed in the presence of transfluthrin intervention system compared to the controls | (45) |
| Fillinger (2023) | - | Passive fabric | Wild strain | | | 68 nights | No info | No info | (46) |
| Wagman (2015) | - | Passive fabric | No info | | | 4 days | No info | No info | (47) |
| Masalu (2020) | - | Passive fabric | Wild strain and  Lab-reared | | | 34 nights | No info | Mortality of the mosquitoes exposed to untreated chairs remained low (5.2% for field-collected *An. arabiensis*, 0.0% for field-collected *An. funestus*, 0.1% for laboratory-reared *An. arabiensis* and 1.1% for laboratory-reared *Ae. aegypti* | (48) |
| Mponzi (2022) | 9.6 x 21 | Passive fabric | Lab-reared  Nulliparous  4 to 9 days old  Starved for 6 hrs | | | 20 nights | No info | No info | (49) |
| Mmbando (2023) | - | Passive fabric | Wild strain | | | 16 nights | - | Mortality of field-collected mosquitoes exposed to TER, TS or both interventions was 56–78% for *An. arabiensis* and 47–74% for *An. funestus*. | (50) |
| Swai (2019) |  | Passive fabric | Wild strain | | | 21 nights | No info | No info | (41) |
| Mmbando (2018) | 9.6 x 2.1 | Passive fabric | Lab-reared  4 to 8 days old nulliparous female  Starved for 12 hrs | | | 75 (15 per treatment) | No info | Mortality was evaluated at each morning when retrieving the traps. Observation was made after 24 h. | (29) |
| Tambwe (2023) | 6 x 6 x 2 | Passive fabric | Lab-reared  3 to 8 days old  *Aedes* -sugar starved for 12 hrs *An.* mosquitoes for 6 hrs | | | 3 days | Knockdown collection began at 45 – 60 min after exposure to intervention | A clear dose–response was observed for mortality at 24hr *An. gambie* had 99% and *An. fenestus* had 100% mortality. | (51) |
| Britch (2020) | - | Passive fabric | Wild strain | | | 6 nights | No info | No info | (52) |
| Fillinger (2023) | - | Passive fabric | Wild strain | | | 68 nights | No info | No info | (46) |
| Yan (2023) | 40 x 3.5 x 4 | Passive fabric | Lab-reared  3 to 5 day old females  Non-blood fed,  Sugar starved 6 hrs | | | 16 nights | Knockdown reached a peak at 75% | Mortality reached a peak of 38.5%, at transfluthrin 20% and significantly decreased with lower concentration | (40) |
| Salazar (2013) | Pu Teuy experimental huts | Passive fabric | Lab-reared,  3 to 5 days old Sugar starved | | | No info | No info | Exposure to transfluthrin at 4x10^-4^ g using 100, 50 or 25% SAC resulted in high mortality rates of Ae. Aegypti cohorts. Mortality ranged from 95- 100% and prevented BGS trap evaluations | (8) |
| Ogoma (2017) | - | Passive fabric | Wild | | | 12 nights | No info | No info | (39) |
| Moreno-Gómez (2021) | - | Spray | Lab-reared,  5 to 10 days old  non-blood-fed | | |  | Knockdown was 100% among the treatment  from primary or secondary exposure to AI | Mosquitoes at 15 min; mortality was 97.5% ± 5.95 after 24 h. No mortality was observed among the control mosquitoes | (53) |
| Bibbs (2021) | - | Spray | Lab reared,  5 to 7 days old, adult, female non-blood-fed | | | 4 weeks | Knockdown after 20 min, as indicated by ataxia, | Mortality after 24 h, as indicated by unresponsiveness to stimulus | (54) |
| Pates (2002) | - | Commercial requiring energy | Wild strain | | | 25 nights | No info | No info | (55) |
| Kitau (2010) | 12.2 x 8.2 | Commercial passive | Lab reared  4 to 9 days old, adult female *Cx. quinquefasciatus* and *An. gambiae* | | | 9 nights | No info | No info | (56) |
| Msangi (2010) | - | Coil | Wild strains  *An. gambiae s.l*  *Cx. quinquefasciatus.* | | | 3 nights | The low level of pyrethrin have been considered to have no knockdown effect unless the concentration of pyrethrin increased. | In all experimental huts, among *Cx.quinquefasciatus* and *An.gambiae s.l* collected, none died after being held for twenty-four hours of observation in provision of 10% sugar solution. | (57) |
| Rapley (2009) | 3.8 x 2.6 x 2.45 | Commercial passive | Lab-reared  *Ae. Aegypti*  4 to 11-day-old, non-blood fed female | | | No Info | Higher proportion of mosquitoes were found in the treated room after the metofluthrin treatment than the coil or control treatments This was a result of the knockdown of mosquitoes entering the treatment room | Mosquito movement and mortality was assessed at the completion of each treatment replicate by tallying both alive and dead mosquitoes in each room, and identifying from which room the mosquitoes were released by recording their marked colour | (58) |
| Revay (2013) | 10 × 30 × 3 | Commercial passive | Female  5 days old *Cx. pipiens*  Starved for 24hrs | | | 8 nights | No Info | No Info | (59) |

**Table S2.** **Study-level insecticide resistance**

| Author year | Country | Study type | | Mosquito Species^+^ | PE (%) from forest plot | Mosquitoes | | Insecticide resistance* | | | | | | |
| --- | --- | --- | --- | --- | --- | --- | --- | --- | --- | --- | --- | --- | --- | --- |
|  |  | **Field** | **Semi-field** |  |  | **Wild** | **Laboratory reared** | **Permethrin** | **Deltamethrin** | **lambda-cyhalothrin** | **Alpha-cypermethrin** | **Bendiocarb** | **Pirimiphos-**  **methyl** | **Reference for resistance data** |
| Lukwa 2008 | Zimbabwe | x |  | *An. gambiae* | 0.92 |  | x | 100 | 100 | 100 |  | 100 |  | (60, 61) |
| Tangena 2018 | Laos | x |  | *Ae. albopictus* | 0.83 | x |  | 100 | 90 |  |  |  |  | (62) |
| Tangena 2018 | Laos | x |  | *An. barbumbrosus, An. barbirostris, An. dirus, An. maculatus, An. epiroticus, An. umbrosus* | 0.83 | x |  |  |  |  |  |  |  |  |
| Tangena 2018 | Laos | x |  | *Cx. vishnui* | 0.83 | x |  |  |  |  |  |  |  |  |
| Maia 2016 | Tanzania | x |  | *An. funestus* s. s | 0.80 | x |  | 11 | 19 | 19 |  | 95 | 100 | (63, 64) |
| Maia 2016 | Tanzania | x |  | *An. gambiae* | 0.80 | x |  | 58 | 59 |  |  | 100 | 100 | (65) |
| Maia 2016 | Tanzania | x |  | *Cx. univittatus* | 0.80 | x |  |  |  |  |  |  |  |  |
| Maia 2016 | Tanzania | x |  | *Cx. quinquefasciatus* | 0.80 | x |  | 100 | 8-88 | 80-87 |  |  | 100 | (66) |
| Oumbouke 2017 | Benin | x |  | *Cx. quinquefasciatus* | 0.73 | x |  | 4-24 | 24-48 |  |  | 60-76 |  | (67) |
| Achee 2012 | Thailand | x |  | *Ae. aegypti* | 0.45 |  | x | 9 | 100 |  | 98 |  |  | (68) |
| Ogoma 2014 | Tanzania |  | x | *An. arabiensis* | 0.38 | x |  | 29-57 | 37-65 | 34 -53 |  | 100 | 98 - 100 | (69) |
| Syafruddin 2014 | Indonesia | x |  | *An. sundaicus* | 0.33 | x |  |  | Resistant |  | Resistant |  | Susceptible | (70) (71) |
| Syafruddin 2014 | Indonesia | x |  | *An. barbirostris* | 0.33 | x |  |  |  |  | Resistant |  |  | (70) (71) |
| Syafruddin 2014 | Indonesia | x |  | *An. maculatus* | 0.33 | x |  |  |  |  |  |  | Susceptible | (70, 71) |
| Syafruddin 2014 | Indonesia | x |  | *An. subpictus s.l.* | 0.33 | x |  |  |  |  | Resistant |  |  | (70, 71) |
| Syafruddin 2014 | Indonesia | x |  | *An. indefinitus, An. vagus, An. annularis, An. aconitus, An. kochi, An. tessellatus* | 0.33 |  |  | 100 |  |  |  |  |  | (Lobo N, pers. comm) |
| Ogoma 2014 | Tanzania | x |  | *An. arabiensis* | 0.30 | x |  | 29-57 | 37-65 | 34 -53 |  | 100 | 98 - 100 | (69) |
| Salazar 2013 | Thailand |  | x | *Ae. aegypti* | -0.03 |  | x | 9 | 100 |  | 98 |  |  | (68) |
| Vajda 2023 | Thailand | x |  | *An. minimus* | 0.99 |  | x | 93 | 100 | 100 | 98 |  |  | (72) |
| Zarella States) 2022 | United States | x |  | *Ae. albopictus, Ae. aegypti* | 0.90 | x |  | 99 | 100 |  |  |  |  | (10) |
| Kawada 2005 | Indonesia | x |  | *An. sundaicus* | 0.88 |  |  |  | Resistant |  | Resistant |  | Susceptible | (70, 71) |
| Kawada 2005 | Indonesia | x |  | *Cx quinquefasciatus* |  |  |  |  |  |  |  |  |  |  |
| McPhatter States of America) 2017 | United States of America |  | x | *Ae. aegypti* | 0.75 |  | x |  |  |  |  |  |  |  |
| Zarella States) 2022 | United States | x |  | *Ae. albopictus* | 0.75 | x | x |  | 99 |  |  | 100 |  | (73) |
| Zarella States) 2022 | United States | x |  | *Ae aegypti* | 0.75 |  | x |  |  |  |  |  |  |  |
| Swai 2023 | Tanzania |  | x | *An. arabiensis* | 0.70 | x |  | 51 | 18 | 45 | 12 |  |  | (Moore S, unpublished.) |
| Stevenson 2018 | Zambia | x |  | *An. gambiae* | 0.32 |  | x | 60 | 60 | 60 | 60 |  |  | (74) (75) |
| Kawada 2008 | Tanzania | x |  | *An. gambiae* | 0.29 | x |  | 95 | 78 |  |  | 99 | 100 | (65) |
| Devine 2021 | Mexico | x |  | *Ae. aegypti* | 0.50 | x | x |  |  |  |  |  |  |  |
| Charlwood 2014 | Cambodia | x |  | *Aedes albopictus* | 0.47 | x |  | 6 | 2 |  |  |  |  | (18) |
| Charlwood 2016 | Cambodia | x |  | *An. minimus* | 0.39 | x |  | >98 | >98 | >98 |  |  |  | (76) |
| Charlwood 2016 | Cambodia | x |  | *An. maculatus, An. hodgkini* | 0.39 | x |  | Possible resistant |  |  |  |  |  | (18) |
| Burton 2023 | Zambia |  | x | *An. gambiae* | 0.39 |  | x |  |  |  |  |  |  |  |
| Charlwood 2017 | Cambodia | x |  | *An. dirus* | 0.37 |  |  | >98 | >98 | >98 |  |  |  | (76) |
| Flores-Mendoza 2022 | Peru | x |  | *An. darlingi* | 0.35 | x |  | Likely susceptible |  |  |  |  |  | [16] |
| Flores-Mendoza 2022 | Peru | x |  | *Cx pedroi, Cx. Corotor* | 0.35 |  |  |  |  |  |  |  |  |  |
| Morrison 2022 | Peru | x |  | *Ae. aegypti* | 0.24 |  | x |  | 43-60 | 21-28 | 2-8 |  | 1-4 | (77) |
| Syafruddin 2020 | Indonesia | x |  | *An. vagus* | 0.18 | x | x | 100 |  |  |  |  |  | (23) |
| Syafruddin 2020 | Indonesia | x |  | *An. aconitus, an. annularis, An. barbirostris, An. flavirostris, An. kochi, An. maculatus, An. subpictus, An. sundaicus, An. tessellatus* | 0.18 | x |  | 94 | 94 | 94 | 94 |  |  | (75) |
| Permana 2022 | Indonesia | x |  | *An. aconitus* | 0.16 | x |  |  | Resistant |  |  |  | Susceptible | (71) (70, 75) |
| Permana 2022 | Indonesia | x |  | *An. kochi* | 0.16 | x |  |  |  |  |  | Resistant |  |  |
| Permana 2022 | Indonesia | x |  | *An. sundaicus* | 0.16 | x |  |  | Resistant |  | Resistant |  | Susceptible |  |
| Permana 2022 | Indonesia | x |  | *An. maculatus* | 0.16 | x |  | 94 | 94 | 94 | 94 |  | susceptible |  |
| Permana 2022 | Indonesia | x |  | *An. flavirostris, An. balabacenisis, An. essellatus, An. sudaicus* | 0.16 | x |  | 94 | 94 | 94 | 94 |  |  | (75) |
| Lloyd 2013 | United States of America | x |  | *Ae. albopictus* | 0.59 | x |  |  | 99 |  |  |  |  | (73) |
| Lloyd 2013 | United States of America | x |  | *Ae. albopictus* | 0.63 | x |  |  | 99 |  |  |  |  | (73) |
| Britch 2021 | United States of America | x |  | *An. quadrimaculatus, Cx. erraticus* | 0.60 |  | x | N/A |  |  |  |  |  |  |
| Frances 2020* | Australia | x |  | *Ae. vigilax* | 0.52 |  |  |  |  |  |  |  | 100 | (78) |
| Dame 2014 | United States of America | x |  | *An. quadrimaculatus, Cx. erraticus* | 0.41 |  |  | N/A |  |  |  |  |  |  |
| Dame 2014 | United States of America | x |  | *An. quadrimaculatus, Cx. erraticus* | 0.04 |  |  | N/A |  |  |  |  |  |  |
| Mmbando 2018 | Tanzania |  | x | *An. arabiensis* | 0.97 | x | x | 19 | 43 | 18 |  | 100 |  |  |
| Ogoma 2012 | Tanzania |  | x | *An. gambiae* | 0.98 |  | x |  |  |  |  |  |  |  |
| Govella 2015 | Tanzania | x |  | *An. arabiensis* | 0.95 |  | x | 19 | 43 | 18 |  | 100 |  | (31) (Moore S, unpublished.) |
| Njoroge 2022 | Kenya |  | x | *An. arabiensis* | 0.92 |  | x |  | 91 |  | 71 |  |  | (79) (Njoroge M, pers comm.) |
| Tambwe 2021 | Tanzania |  | x | *Ae. aegypti* | 0.88 |  | x | 100 | 100 | 100 | 100 |  | 74 | (Moore S, unpublished) |
| Masalu 2018 | Tanzania | x |  | *An. arabiensis* | 0.88 |  | x | 38-60 | 90-78 | 22-69 |  | 100 | 100 | (80) |
| Masalu 2018 | Tanzania | x |  | *An. funestus s.s.^+^* | 0.88 |  |  | 11 | 19 | 19 |  | 95 | 100 | (81) |
| Mmbando 2017 | Tanzania | x |  | *An. gambiae s.s.^+^* | 0.75 |  |  | 100 | 100 | 100 |  | 100 | 100 | (80) |
| Mmbando 2017 | Tanzania | x |  | *An. funestus s.s.^+^* | 0.75 |  |  | 11 | 19 | 19 |  | 95 | 100 | (81) |
| Masalu 2017 | Tanzania | x |  | *An. arabiensis* | 0.84 |  |  | 38-60 | 90-78 | 22-69 |  | 100 | 100 | (80) |
| Masalu 2017 | Tanzania | x |  | *Cx. species* | 0.84 |  |  | 100 | 8-88 | 80-87 |  |  | 100 | (63) |
| Njoroge 2021 | Kenya |  | x | *An. arabiensis* | 0.83 |  |  |  | 91 |  |  | 96 |  | (Njoroge M, pers comm.) |
| Andrés 2015 | Tanzania |  | x | *An. arabiensis* | 0.83 |  |  | 19 | 43 | 18 |  | 100 |  | (Moore S, unpublished) |
| Andrés 2015 | Tanzania |  | x | *An. gambiae* | 0.83 |  |  | 100 | 100 | 100 |  | 100 |  | (Moore S, unpublished) |
| Njoroge 2022 | Kenya |  | x | *An. arabiensis* | 0.78 |  | x |  | 91 |  | 71 |  |  | (79) (Njoroge M, pers comm.) |
| Ogoma 2017 | Tanzania | x |  | *An. arabiensis* | 0.75 |  |  | 79 | 79 | 79 | 79 |  |  | (75) |
| Ogoma 2017 | Tanzania | x |  | *An. funestus* | 0.75 |  |  | 79 | 79 | 79 | 79 |  |  | (75) |
| Ogoma 2017 | Tanzania | x |  | *An. coustani* | 0.75 |  |  |  |  |  |  |  |  |  |
| Yan 2023 | Thailand |  | x | *An. minimus* | 0.53 |  | x | 93 | 100 | 100 | 98 |  |  | (72, 82) |
| Swai 2019 | Tanzania | x |  | *An. arabiensis* | 0.58 | x |  | 38-60 | 90-78 | 22-69 |  | 100 | 100 | (80) |
| Swai 2019 | Tanzania | x |  | *An. funestus* | 0.58 | x |  | 11 | 19 | 19 |  | 95 | 100 | (81) |
| Sukkanon 2021 | Thailand |  | x | *An. harrisoni* | 0.66 | x |  |  |  |  |  |  |  |  |
| Sangoro 2020 | Tanzania | x | x | *An. arabiensis* | 0.66 |  | x | 11 | 18 | 13 | 15 |  |  | (Moore S, unpublished.) |
| Sangoro 2020 | Tanzania | x | x | *An. funestus* | 0.66 |  | x | 11 | 19 | 19 |  | 95 | 100 | (81) |
| Tambwe 2020 | Tanzania |  | x | *Ae. aegypti* | 0.61 |  | x | 100 | 100 |  | 100 |  |  | (Moore S, unpublished.) |
| Tambwe 2021 | Tanzania |  | x | *Ae. aegypti* | 0.60 |  | x | 100 | 100 | 100 | 100 |  | 74 | (Moore S, unpublished.) |
| Tambwe 2021 | Tanzania |  | x | *An. gambiae (Ifakara strain)* | 0.60 |  | x | 79 | 100 | 100 | 100 | 100 | 100 | (Moore S, unpublished.) |
| Tambwe 2021 | Tanzania |  | x | *An. arabiensis (Mbita strain)* | 0.60 |  | x |  | 91 |  |  | 96 |  | (Njoroge M, pers comm.) |
| Tambwe 2021 | Tanzania |  | x | *An. arabiensis (Kingani strain)* | 0.60 |  | x | 10 | 19 | 17 | 14 | 100 | 100 | (Moore S, unpublished.) |
| Wagman 2015 | Belize | x |  | *An. albimanus* | 0.51 |  |  | 100 | 97-100 |  |  |  |  | (83), (Wagman J, pers. Comm) |
| Wagman 2015 | Belize | x |  | *An. vestitipennis* | 0.51 |  |  | 97-100 | 97-100 |  |  |  |  | (Wagman J, pers. Comm) |
| Masalu 2020 | Tanzania | x |  | *An. arabiensis* | 0.52 | x |  | 38-60 | 90-78 | 22-69 |  | 100 | 100 | (80) |
| Masalu 2020 | Tanzania | x |  | *An. funestus* | 0.52 | x |  | 11 | 19 | 19 |  | 95 | 100 | (81) |
| Mponzi 2022 | Tanzania |  | x | *Ae. aegypti* | 0.50 |  | x |  |  |  |  |  |  |  |
| Mponzi 2022 | Tanzania |  | x | *An. arabiensis* | 0.50 |  | x | 79 | 79 | 79 | 79 |  |  | (75) |
| Mmbando 2023 | Tanzania | x |  | *An. arabiensis* | 0.43 | x |  | 29-57 | 37-65 | 34 - 53 |  | 100 | 98 -100 | (50, 69) |
| Mmbando 2023 | Tanzania | x |  | *An. funestus* | 0.43 | x |  | 11 | 19 | 19 |  | 95 | 100 | (81) |
| Tambwe 2023 | Tanzania |  | x | *An. gambiae* | 0.44 |  | x | 100 | 100 | 100 | 100 | 100 | 100 | (Moore S, unpublished.) |
| Tambwe 2023 | Tanzania |  | x | *An. funestus* | 0.44 |  | x | 60 | 78 | 66 | 72 | 94 | 100 | (Moore S, unpublished.) |
| Britch 2020 | United States | x |  | *Cx. quinquefasciatus* | 0.30 | x |  | High resistance |  |  |  |  |  | (84-86) |
| Britch 2020 | United States | x |  | *Ae. dorsalis* | 0.43 | x |  |  |  |  |  |  |  |  |
| Britch 2020 | United States | x |  | *An. hermsi* | 0.43 | x |  |  |  |  |  |  |  |  |
| Britch 2020 | United States | x |  | *Culiseta inorta* | 0.43 | x |  |  |  |  |  |  |  |  |
| Britch 2020 | United States | x |  | *Cx. Erythrothorax* | 0.43 | x |  |  |  |  |  |  |  |  |
| Britch 2020 | United States | x |  | *Cx. quinquefasciatus* | 0.43 | x |  |  |  |  |  |  |  |  |
| Britch 2020 | United States | x |  | *Cx. Tarsalis* | 0.43 | x |  |  |  |  |  |  |  |  |
| Fillinger 2023 | Kenya | x |  | *An. arabiensis* | 0.34 | x |  | 20 | 8 |  |  |  |  | (87, 88) |
| Fillinger 2023 | Kenya | x |  | *An. funestus* | 0.34 |  |  | <90 | <90 |  |  |  |  | (89) |
| Fillinger 2023 | Kenya | x |  | *Cx.*  *Mansonia* | 0.34 |  |  |  |  |  |  |  |  |  |
| Tangena 2018 | Laos | x |  | *Ae. albopictus* | 0.31 |  | x |  |  |  |  | 99 |  | (90) |
| Tangena 2018 | Laos | x |  | *An. barbumbrosus, An. barbirostris, An. dirus, An. maculatus, An. epiroticus, An. umbrosus* | 0.31 |  | x |  |  |  |  |  |  |  |
| Sukkanon 2021 | Thailand | x |  | *An. minimus* | 0.21 |  | x |  |  |  |  |  |  |  |
| Sukkanon 2021 | Thailand | x |  | *Ae. aegypti* | 0.21 |  | x |  |  |  |  |  |  |  |
| Moreno-Gómez 2021 | Italy | x |  | *Ae. albopictus* | 0.73 |  | x | 98 |  |  | 100 |  |  | (91) |
| Bibbs 2021 | United States | x |  | *Ae. albopictus* | 0.33 |  | x | 95 |  |  |  |  |  | (92) |

* Resistance reported as % knock down after 60 minutes or % mortality after 24 hours

^+^ Species details from personal communications

**Colour code for % mortality**

White, no number = data unavailable

White with number, confirmed susceptible to insecticide

Light grey with number, mortality >= 90% but <98%, possible resistance to insecticide

Dark grey with number, mortality <90%, confirmed resistance

**Table S3. Risk of bias heat map**

| **Author & (year)** | **Randomization for spatial and temporal heterogeneity** | | **Blinding** | | | | **Mosquito conditions (semi-field only)** | **Funder involve-ment** |
| --- | --- | --- | --- | --- | --- | --- | --- | --- |
|  | **Study design** | **CRCTs only: baseline characteristics** | **Participants** | **Investigator** | **Statistician** | **Outcome reporting** |  |  |
| Lukwa, Nzira (2008) |  |  |  |  |  |  |  |  |
| Tangena, J-A A. 2018 |  |  |  |  |  |  |  |  |
| Maia 2016 |  |  |  |  |  |  |  |  |
| Oumbouke 2017 | \|  \| \| --- \| |  |  |  |  |  |  |  |
| Achee, N. 2012 |  |  |  |  |  |  |  |  |
| Ogoma 2014 |  |  |  |  |  |  |  |  |
| Syafruddin 2014 |  |  |  |  |  |  |  |  |
| Salazar2013 |  |  |  |  |  |  |  |  |
| Vadja 2023 |  |  |  |  |  |  |  |  |
| Zarella 2022 |  |  |  |  |  |  |  |  |
| Kawada 2005 |  |  |  |  |  |  |  |  |
| McPhatter 2017 |  |  |  |  |  |  |  |  |
| Zarella 2022 |  |  |  |  |  |  |  |  |
| Swai 2023 |  |  |  |  |  |  |  |  |
| Stevenson 2018 |  |  |  |  |  |  |  |  |
| Kawada 2008 |  |  |  |  |  |  |  |  |
| Devine 2021 |  |  |  |  |  |  |  |  |
| Charlwood 2014 |  |  |  |  |  |  |  |  |
| Charlwood 2016 |  |  |  |  |  |  |  |  |
| Burton 2023 |  |  |  |  |  |  |  |  |
| Charlwood 2017 |  |  |  |  |  |  |  |  |
| Flores-Mendoza 2022 |  |  |  |  |  |  |  |  |
| Morrison 2022 |  |  | 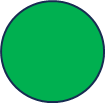 |  |  |  |  |  |
| Syafruddin 2020 |  |  |  |  |  |  |  |  |
| Permana 2022 |  |  |  |  |  |  |  |  |
| Kawada 2008 |  |  |  |  |  |  |  |  |
| Stevenson 2018 |  |  |  |  |  |  |  |  |
| Lloyd 2013 |  |  |  |  |  |  |  |  |
| Britch 2021 |  |  |  |  |  |  |  |  |
| Frances 2020 |  |  |  |  |  |  |  |  |
| Dame 2014 |  |  |  |  |  |  |  |  |
| Mmbando 2018 |  |  |  |  |  |  |  |  |
| Ogoma 2012 |  |  |  |  |  |  |  |  |
| Govella 2015 |  |  |  |  |  |  |  |  |
| Njoroge 2022 |  |  |  |  |  |  |  |  |
| Tambwe 2021 |  |  |  |  |  |  |  |  |
| Masalu 2018 |  |  |  |  |  |  |  |  |
| Mmbando 2017 |  |  |  |  |  |  |  |  |
| Masalu 2017 |  |  |  |  |  |  |  |  |
| Njoroge 2021 |  |  |  |  |  |  |  |  |
| Andrés 2015 |  |  |  |  |  |  |  |  |
| Njoroge 2022 |  |  |  |  |  |  |  |  |
| Ogoma 2017 |  |  |  |  |  |  |  |  |
| Yan 2023 |  |  |  |  |  |  |  |  |
| Swai 2019 |  |  |  |  |  |  |  |  |
| Masalu 2018 |  |  |  |  |  |  |  |  |
| Sukkanon 2021 |  |  |  |  |  |  |  |  |
| Sangoro 2020 |  |  |  |  |  |  |  |  |
| Tambwe 2020 |  |  |  |  |  |  |  |  |
| Fillinger 2023 |  |  |  |  |  |  |  |  |
| Wagman 2015 |  |  |  |  |  |  |  |  |
| Masalu 2020 |  |  |  |  |  |  |  |  |
| Mponzi 2022 |  |  |  |  |  |  |  |  |
| Mmbando 2023 |  |  |  |  |  |  |  |  |
| Tambwe 2023 |  |  |  |  |  |  |  |  |
| Britch 2020 |  |  |  |  |  |  |  |  |
| Moreno-Gómez 2021 |  |  |  |  |  |  |  |  |
| Bibbs 2021 |  |  |  |  |  |  |  |  |

Notes: For funder involvement, three papers had some concerns: 1) Swai 2023, M. Chura, and entomologist from SCJ was a co-author. 2) Flores-Mendoza 2023 and 3) Stevenson 2018, N. Elman was senior author, he is the CEO and founder of Gearjump, for both papers the authors declare no conflict of interest.

**Legend:**

High
Some concern
Unclear
Low

**References**

1. Lukwa N, Chiwade T. Lack of insecticidal effect of mosquito coils containing either metofluthrin or esbiothrin on Anopheles gambiae sensu lato mosquitoes. Trop Biomed. 2008;25(3):191-5.

2. Tangena J-AA, Thammavong P, Chonephetsarath S, Logan JG, Brey PT, Lindsay SW. Field evaluation of personal protection methods against outdoor-biting mosquitoes in Lao PDR. Parasites & Vectors. 2018;11(1):661.

3. Maia MF, Kreppel K, Mbeyela E, Roman D, Mayagaya V, Lobo NF, et al. A crossover study to evaluate the diversion of malaria vectors in a community with incomplete coverage of spatial repellents in the Kilombero Valley, Tanzania. Parasites & Vectors. 2016;9(1):451.

4. Oumbouke WA, Fongnikin A, Soukou KB, Moore SJ, N'Guessan R. Relative performance of indoor vector control interventions in the Ifakara and the West African experimental huts. Parasit Vectors. 2017;10(1):432.

5. Achee N, Masuoka P, Smith P, Martin N, Chareonviryiphap T, Polsomboon S, et al. Identifying the effective concentration for spatial repellency of the dengue vector Aedes aegypti. Parasites & Vectors. 2012;5(1):300.

6. Ogoma SB, Lorenz LM, Ngonyani H, Sangusangu R, Kitumbukile M, Kilalangongono M, et al. An experimental hut study to quantify the effect of DDT and airborne pyrethroids on entomological parameters of malaria transmission. Malaria Journal. 2014;13(1):131.

7. Syafruddin D, Bangs MJ, Sidik D, Elyazar I, Asih PB, Chan K, et al. Impact of a Spatial Repellent on Malaria Incidence in Two Villages in Sumba, Indonesia. The American Society of Tropical Medicine and Hygiene. 2014;91(6):1079-87.

8. Salazar FV, Achee NL, Grieco JP, Prabaripai A, Ojo TA, Eisen L, et al. Effect of Aedes aegypti exposure to spatial repellent chemicals on BG-Sentinel™ trap catches. Parasites & Vectors. 2013;6(1):145.

9. Vajda É A, Saeung M, Ross A, McIver DJ, Tatarsky A, Moore SJ, et al. A semi-field evaluation in Thailand of the use of human landing catches (HLC) versus human-baited double net trap (HDN) for assessing the impact of a volatile pyrethroid spatial repellent and pyrethroid-treated clothing on Anopheles minimus landing. Malar J. 2023;22(1):202.

10. Zarella O, Ekwomadu U, Romer Y, Kirstein OD, Che-Mendoza A, González-Olvera G, et al. Experimental evaluation of a metofluthrin passive emanator against Aedes albopictus. PLoS One. 2022;17(5):e0267278.

11. Kawada H, Maekawa Y, Takagi M. Field trial on the spatial repellency of metofluthrin-impregnated plastic strips for mosquitoes in shelters without walls (beruga) in Lombok, Indonesia. J Vector Ecol. 2005;30(2):181-5.

12. McPhatter LP, Mischler PD, Webb MZ, Chauhan K, Lindroth EJ, Richardson AG, et al. Laboratory and semi-field evaluations of two (transfluthrin) spatial repellent devices against Aedes aegypti (L.) (Diptera: Culicidae). US Army Med Dep J. 2017(1-17):13-22.

13. Swai JK, Soto AC, Ntabaliba WS, Kibondo UA, Ngonyani HA, Mseka AP, et al. Efficacy of the spatial repellent product Mosquito Shield™ against wild pyrethroid-resistant Anopheles arabiensis in south-eastern Tanzania. Malaria Journal. 2023;22(1):249.

14. Stevenson JC, Simubali L, Mudenda T, Cardol E, Bernier UR, Vazquez AA, et al. Controlled release spatial repellent devices (CRDs) as novel tools against malaria transmission: a semi-field study in Macha, Zambia. Malaria Journal. 2018;17(1):437.

15. Kawada H, Temu EA, Minjas JN, Matsumoto O, Iwasaki T, Takagi M. Field evaluation of spatial repellency of metofluthrin-impregnated plastic strips against Anopheles gambiae complex in Bagamoyo, coastal Tanzania. J Am Mosq Control Assoc. 2008;24(3):404-9.

16. Devine GJ, Vazquez-Prokopec GM, Bibiano-Marín W, Pavia-Ruz N, Che-Mendoza A, Medina-Barreiro A, et al. The entomological impact of passive metofluthrin emanators against indoor Aedes aegypti: A randomized field trial. PLOS Neglected Tropical Diseases. 2021;15(1):e0009036.

17. Charlwood JD, Tomás EVE, Kelly-Hope L, Briët OJT. Evidence of an ‘invitation’ effect in feeding sylvatic Stegomyia albopicta from Cambodia. Parasites & Vectors. 2014;7(1):324.

18. Charlwood JD, Nenhep S, Protopopoff N, Sovannaroth S, Morgan JC, Hemingway J. Effects of the spatial repellent metofluthrin on landing rates of outdoor biting anophelines in Cambodia, Southeast Asia. Med Vet Entomol. 2016;30(2):229-34.

19. Burton TA, Kabinga LH, Simubali L, Hayre Q, Moore SJ, Stevenson JC, et al. Semi-field evaluation of a volatile transfluthrin-based intervention reveals efficacy as a spatial repellent and evidence of other modes of action. PLOS ONE. 2023;18(5):e0285501.

20. Charlwood JD, Hall T, Nenhep S, Rippon E, Branca-Lopes A, Steen K, et al. Spatial repellents and malaria transmission in an endemic area of Cambodia with high mosquito net usage. Malariaworld J. 2017;8:11.

21. Flores-Mendoza C, López-Sifuentes VM, Vásquez GM, Stoops CA, Fisher ML, Bernier UR, et al. Field Evaluation of Novel Spatial Repellent Controlled Release Devices (CRDs) against Mosquitoes in an Outdoor Setting in the Northern Peruvian Amazon. Trop Med Infect Dis. 2022;7(11).

22. Morrison AC, Reiner RC, Jr., Elson WH, Astete H, Guevara C, Del Aguila C, et al. Efficacy of a spatial repellent for control of Aedes-borne virus transmission: A cluster-randomized trial in Iquitos, Peru. Proc Natl Acad Sci U S A. 2022;119(26):e2118283119.

23. Syafruddin D, Asih PBS, Rozi IE, Permana DH, Nur Hidayati AP, Syahrani L, et al. Efficacy of a Spatial Repellent for Control of Malaria in Indonesia: A Cluster-Randomized Controlled Trial. Am J Trop Med Hyg. 2020;103(1):344-58.

24. Permana DH, Zubaidah S, Syahrani L, Asih PBS, Syafruddin D, Rozi IE, et al. Impact of a spatial repellent product on Anopheles and non-Anopheles mosquitoes in Sumba, Indonesia. Malaria Journal. 2022;21(1):166.

25. Lloyd AM, Farooq M, Diclaro JW, Kline DL, Estep AS. Field evaluation of commercial off-the-shelf spatial repellents against the Asian tiger mosquito, Aedes Albopictus (Skuse), and the potential for use during deployment. US Army Med Dep J. 2013:80-6.

26. Britch SC, Dame DA, Meisch MV, Kline DL, Walker TW, Allan SA, et al. Spatial Repellents Protect Small Perimeters from Riceland Mosquitoes in a Warm-Humid Environment. Journal of the American Mosquito Control Association. 2021;37(1):41-5.

27. Frances SP, Rowcliffe KL, MacKenzie DO. Field Effectiveness of a Metofluthrin Fan-Based Emanator and Deet as Repellents Against Aedes vigilax in Southeast Queensland, Australia1. J Am Mosq Control Assoc. 2020;36(2):120-2.

28. Dame DA, Meisch MV, Lewis CN, Kline DL, Clark GG. Field evaluation of four spatial repellent devices against Arkansas rice-land mosquitoes. J Am Mosq Control Assoc. 2014;30(1):31-6.

29. Mmbando AS, Ngowo H, Limwagu A, Kilalangongono M, Kifungo K, Okumu FO. Eave ribbons treated with the spatial repellent, transfluthrin, can effectively protect against indoor-biting and outdoor-biting malaria mosquitoes. Malaria Journal. 2018;17(1):368.

30. Ogoma SB, Ngonyani H, Simfukwe ET, Mseka A, Moore J, Killeen GF. Spatial repellency of transfluthrin-treated hessian strips against laboratory-reared Anopheles arabiensis mosquitoes in a semi-field tunnel cage. Parasit Vectors. 2012;5:54.

31. Govella NJ, Ogoma SB, Paliga J, Chaki PP, Killeen G. Impregnating hessian strips with the volatile pyrethroid transfluthrin prevents outdoor exposure to vectors of malaria and lymphatic filariasis in urban Dar es Salaam, Tanzania. Parasites & Vectors. 2015;8(1):322.

32. Njoroge MM, Hiscox A, Saddler A, Takken W, van Loon JJA, Fillinger U. Less is more: repellent-treated fabric strips as a substitute for full screening of open eave gaps for indoor and outdoor protection from malaria mosquito bites. Parasites & Vectors. 2022;15(1):259.

33. Tambwe MM, Saddler A, Kibondo UA, Mashauri R, Kreppel KS, Govella NJ, et al. Semi-field evaluation of the exposure-free mosquito electrocuting trap and BG-Sentinel trap as an alternative to the human landing catch for measuring the efficacy of transfluthrin emanators against Aedes aegypti. Parasit Vectors. 2021;14(1):265.

34. Masalu JP, Okumu FO, Mmbando AS, Sikulu-Lord MT, Ogoma SB. Potential benefits of combining transfluthrin-treated sisal products and long-lasting insecticidal nets for controlling indoor-biting malaria vectors. Parasit Vectors. 2018;11(1):231.

35. Mmbando AS, Ngowo HS, Kilalangongono M, Abbas S, Matowo NS, Moore SJ, et al. Small-scale field evaluation of push-pull system against early- and outdoor-biting malaria mosquitoes in an area of high pyrethroid resistance in Tanzania. Wellcome Open Res. 2017;2:112.

36. Masalu JP, Finda M, Okumu FO, Minja EG, Mmbando AS, Sikulu-Lord MT, et al. Efficacy and user acceptability of transfluthrin-treated sisal and hessian decorations for protecting against mosquito bites in outdoor bars. Parasites & Vectors. 2017;10(1):197.

37. Njoroge MM, Fillinger U, Saddler A, Moore S, Takken W, van Loon JJA, et al. Evaluating putative repellent ‘push’ and attractive ‘pull’ components for manipulating the odour orientation of host-seeking malaria vectors in the peri-domestic space. Parasites & Vectors. 2021;14(1):42.

38. Andrés M, Lorenz LM, Mbeleya E, Moore SJ. Modified mosquito landing boxes dispensing transfluthrin provide effective protection against Anopheles arabiensis mosquitoes under simulated outdoor conditions in a semi-field system. Malar J. 2015;14:255.

39. Ogoma SB, Mmando AS, Swai JK, Horstmann S, Malone D, Killeen GF. A low technology emanator treated with the volatile pyrethroid transfluthrin confers long term protection against outdoor biting vectors of lymphatic filariasis, arboviruses and malaria. PLoS Negl Trop Dis. 2017;11(4):e0005455.

40. Yan C, Hii J, Ngoen-Klan R, Ahebwa A, Saeung M, Chareonviriyaphap T. The effect of transfluthrin-treated jute and cotton emanator vests on human landing and fecundity of Anopheles minimus in Thailand. Acta Tropica. 2023;242:106904.

41. Swai JK, Mmbando AS, Ngowo HS, Odufuwa OG, Finda MF, Mponzi W, et al. Protecting migratory farmers in rural Tanzania using eave ribbons treated with the spatial mosquito repellent, transfluthrin. Malaria Journal. 2019;18(1):414.

42. Sukkanon C, Tisgratog R, Muenworn V, Bangs MJ, Hii J, Chareonviriyaphap T. Field Evaluation of a Spatial Repellent Emanation Vest for Personal Protection Against Outdoor Biting Mosquitoes. J Med Entomol. 2021;58(2):756-66.

43. Sangoro OP, Gavana T, Finda M, Mponzi W, Hape E, Limwagu A, et al. Evaluation of personal protection afforded by repellent-treated sandals against mosquito bites in south-eastern Tanzania. Malar J. 2020;19(1):148.

44. Tambwe MM, Moore SJ, Chilumba H, Swai JK, Moore JD, Stica C, et al. Semi-field evaluation of freestanding transfluthrin passive emanators and the BG sentinel trap as a “push-pull control strategy” against Aedes aegypti mosquitoes. Parasites & Vectors. 2020;13(1):392.

45. Tambwe MM, Moore S, Hofer L, Kibondo UA, Saddler A. Transfluthrin eave-positioned targeted insecticide (EPTI) reduces human landing rate (HLR) of pyrethroid resistant and susceptible malaria vectors in a semi-field simulated peridomestic space. Malar J. 2021;20(1):357.

46. Fillinger U, Denz A, Njoroge MM, Tambwe MM, Takken W, van Loon JJA, et al. A randomized, double-blind placebo-control study assessing the protective efficacy of an odour-based 'push-pull' malaria vector control strategy in reducing human-vector contact. Sci Rep. 2023;13(1):11197.

47. Wagman JM, Grieco JP, Bautista K, Polanco J, Briceño I, King R, et al. The field evaluation of a push-pull system to control malaria vectors in northern Belize, Central America. Malar J. 2015;14:184.

48. Masalu JP, Finda M, Killeen GF, Ngowo HS, Pinda PG, Okumu FO. Creating mosquito-free outdoor spaces using transfluthrin-treated chairs and ribbons. Malaria Journal. 2020;19(1):109.

49. Mponzi WP, Swai JK, Kaindoa EW, Kifungo K, Eiras AE, Batista EPA, et al. Observing the distribution of mosquito bites on humans to inform personal protection measures against malaria and dengue vectors. PLOS ONE. 2022;17(7):e0271833.

50. Mmbando AS, Mponzi WP, Ngowo HS, Kifungo K, Kasubiri R, Njalambaha RM, et al. Small-scale field evaluation of transfluthrin-treated eave ribbons and sandals for the control of malaria vectors in rural Tanzania. Malaria Journal. 2023;22(1):43.

51. Tambwe MM, Kibondo UA, Odufuwa OG, Moore J, Mpelepele A, Mashauri R, et al. Human landing catches provide a useful measure of protective efficacy for the evaluation of volatile pyrethroid spatial repellents. Parasit Vectors. 2023;16(1):90.

52. Britch SC, Linthicum KJ, Kline DL, Aldridge RL, Golden FV, Wittie J, et al. Transfluthrin Spatial Repellent on US Military Materials Reduces Culex tarsalis Incursion in a Desert Environment. J Am Mosq Control Assoc. 2020;36(1):37-42.

53. Moreno-Gómez M, Miranda MA, Bueno-Marí R. To Kill or to Repel Mosquitoes? Exploring Two Strategies for Protecting Humans and Reducing Vector-Borne Disease Risks by Using Pyrethroids as Spatial Repellents. Pathogens. 2021;10(9).

54. Bibbs CS, Kaufman PE, Xue RD. Adulticidal Efficacy and Sublethal Effects of Metofluthrin in Residual Insecticide Blends Against Wild Aedes albopictus (Diptera: Culicidae). J Econ Entomol. 2021;114(2):928-36.

55. Pates HV, Line JD, Keto AJ, Miller JE. Personal protection against mosquitoes in Dar es Salaam, Tanzania, by using a kerosene oil lamp to vaporize transfluthrin. Med Vet Entomol. 2002;16(3):277-84.

56. Kitau J, Pates H, Rwegoshora TR, Rwegoshora D, Matowo J, Kweka EJ, et al. The effect of Mosquito Magnet Liberty Plus trap on the human mosquito biting rate under semi-field conditions. J Am Mosq Control Assoc. 2010;26(3):287-94.

57. Msangi S, Mwang'onde BJ, Mahande AM, Kweka EJ. Field evaluation of the bio-efficacy of three pyrethroid based coils against wild populations of anthropophilic mosquitoes in Northern Tanzania. Journal of Global Infectious Diseases. 2010;2(2).

58. Rapley LP, Russell RC, Montgomery BL, Ritchie SA. The effects of sustained release metofluthrin on the biting, movement, and mortality of Aedes aegypti in a domestic setting. Am J Trop Med Hyg. 2009;81(1):94-9.

59. Revay EE, Junnila A, Xue RD, Kline DL, Bernier UR, Kravchenko VD, et al. Evaluation of commercial products for personal protection against mosquitoes. Acta Trop. 2013;125(2):226-30.

60. Lukwa N, Sande S, Makuwaza A, Chiwade T, Netsa M, Asamoa K, et al. Nationwide assessment of insecticide susceptibility in Anopheles gambiae populations from Zimbabwe. Malaria Journal. 2014;13(1):408.

61. N. Lukwa SS, 2 P. Munosiyei,3 M. Zimba4. <Insecticide_susceptibility_tests_conduct.pdf>.

62. Tangena J-AA. The risk of vector-borne disease exposure in rubber plantations of northern Lao PDR: Durham University; 2016.

63. Matowo NS, Abbasi S, Munhenga G, Tanner M, Mapua SA, Oullo D, et al. Fine-scale spatial and temporal variations in insecticide resistance in Culex pipiens complex mosquitoes in rural south-eastern Tanzania. Parasites & vectors. 2019;12:1-13.

64. Lwetoijera DW, Harris C, Kiware SS, Dongus S, Devine GJ, McCall PJ, et al. Increasing role of Anopheles funestus and Anopheles arabiensis in malaria transmission in the Kilombero Valley, Tanzania. Malaria journal. 2014;13:1-10.

65. Kisinza WN, Nkya TE, Kabula B, Overgaard HJ, Massue DJ, Mageni Z, et al. Multiple insecticide resistance in Anopheles gambiae from Tanzania: a major concern for malaria vector control. Malaria Journal. 2017;16(1):439.

66. Matowo NS, Abbasi S, Munhenga G, Tanner M, Mapua SA, Oullo D, et al. Fine-scale spatial and temporal variations in insecticide resistance in Culex pipiens complex mosquitoes in rural south-eastern Tanzania. Parasites & Vectors. 2019;12(1):413.

67. Yadouléton A, Badirou K, Agbanrin R, Jöst H, Attolou R, Srinivasan R, et al. Insecticide resistance status in Culex quinquefasciatus in Benin. Parasit Vectors. 2015;8:17.

68. Thanispong K, Sathantriphop S, Chareonviriyaphap T. Insecticide resistance of Aedes aegypti and Culex quinquefasciatus in Thailand. Journal of Pesticide Science. 2008;33(4):351-6.

69. Matiya DJ, Philbert AB, Kidima WB, Kaaya R, Matowo JJ. Susceptibility status of the malaria vector, Anopheles arabiensis to insecticides used in vector-borne diseases control in areas with heterogeneous sources of pollutants in South-East Tanzania. Transactions of the Royal Society of South Africa. 2022;77(3):195-205.

70. Syafruddin D, Hidayati APN, Asih PBS, Hawley WA, Sukowati S, Lobo NF. Detection of 1014F kdr mutation in four major Anopheline malaria vectors in Indonesia. Malaria Journal. 2010;9(1):315.

71. Elyazar IR, Sinka ME, Gething PW, Tarmidzi SN, Surya A, Kusriastuti R, et al. The distribution and bionomics of anopheles malaria vector mosquitoes in Indonesia. Adv Parasitol. 2013;83:173-266.

72. Thanispong K, Sathantriphop S, Tisgratog R, Tainchum K, Sukkanon C, Bangs MJ, et al. Optimal discriminating concentrations of six synthetic Pyrethroids for monitoring insecticide susceptibility in Anopheles minimus (Diptera: Culicidae), a primary malaria vector in Thailand. Journal of economic entomology. 2018;111(5):2375-82.

73. Marcombe S, Farajollahi A, Healy SP, Clark GG, Fonseca DM. Insecticide Resistance Status of United States Populations of Aedes albopictus and Mechanisms Involved. PLOS ONE. 2014;9(7):e101992.

74. Chanda E, Hemingway J, Kleinschmidt I, Rehman AM, Ramdeen V, Phiri FN, et al. Insecticide Resistance and the Future of Malaria Control in Zambia. PLOS ONE. 2011;6(9):e24336.

75. WHO. WHO global database on insecticide resistance in malaria vectors 2024 [Available from: <https://www.who.int/teams/global-malaria-programme/prevention/vector-control/global-database-on-insecticide-resistance-in-malaria-vectors>.

76. Van Bortel W, Trung HD, Thuan LK, Sochantha T, Socheat D, Sumrandee C, et al. The insecticide resistance status of malaria vectors in the Mekong region. Malaria Journal. 2008;7(1):102.

77. Pinto J, Palomino M, Mendoza-Uribe L, Sinti C, Liebman KA, Lenhart A. Susceptibility to insecticides and resistance mechanisms in three populations of Aedes aegypti from Peru. Parasites & Vectors. 2019;12(1):494.

78. Brown MD, Thomas D, Mason P, Greenwood JG, Kay BH. Laboratory and field evaluation of the efficacy of four insecticides for Aedes vigilax (Diptera: Culicidae) and toxicity to the nontarget shrimp Leander tenuicornis (Decapoda: Palaemonidae). Journal of economic entomology. 1999;92(5):1045-51.

79. Njoroge MM, Hiscox A, Saddler A, Takken W, van Loon JJ, Fillinger U. Less is more: repellent-treated fabric strips as a substitute for full screening of open eave gaps for indoor and outdoor protection from malaria mosquito bites. Parasites & Vectors. 2022;15(1):259.

80. Matowo NS, Munhenga G, Tanner M, Coetzee M, Feringa WF, Ngowo HS, et al. Fine-scale spatial and temporal heterogeneities in insecticide resistance profiles of the malaria vector, Anopheles arabiensis in rural south-eastern Tanzania. Wellcome Open Res. 2017;2:96.

81. Kaindoa EW, Matowo NS, Ngowo HS, Mkandawile G, Mmbando A, Finda M, et al. Interventions that effectively target Anopheles funestus mosquitoes could significantly improve control of persistent malaria transmission in south–eastern Tanzania. PloS one. 2017;12(5):e0177807.

82. Chaumeau V, Cerqueira D, Zadrozny J, Kittiphanakun P, Andolina C, Chareonviriyaphap T, et al. Insecticide resistance in malaria vectors along the Thailand-Myanmar border. Parasites & Vectors. 2017;10(1):165.

83. Dusfour I, Achee NL, Briceno I, King R, Grieco JP. Comparative data on the insecticide resistance of Anopheles albimanus in relation to agricultural practices in northern Belize, CA. Journal of Pest Science. 2009;83(1):41-6.

84. Esterly AT, Alemayehu D, Rusmisel B, Busam J, Shelton TL, Sebay T, et al. Culex erythrothorax (Diptera: Culicidae): Activity periods, insecticide susceptibility and control in California (USA). PLOS ONE. 2020;15(7):e0228835.

85. Unlu I, Buckner EA, Medina J, Vasquez C, Cabrera A, Romero-Weaver AL, et al. Insecticide resistance of Miami-Dade Culex quinquefasciatus populations and initial field efficacy of a new resistance-breaking adulticide formulation. PLOS ONE. 2024;19(2):e0296046.

86. Tsecouras JC, Thiemann TC, Hung KY, Henke JA, Gerry AC. Prevalence of Permethrin Resistance in Culex Tarsalis Populations in Southern California. Journal of the American Mosquito Control Association. 2023;39(4):236-42.

87. Kawada H, Dida GO, Ohashi K, Komagata O, Kasai S, Tomita T, et al. Multimodal Pyrethroid Resistance in Malaria Vectors, Anopheles gambiae s.s., Anopheles arabiensis, and Anopheles funestus s.s. in Western Kenya. PLOS ONE. 2011;6(8):e22574.

88. Mulamba C, Riveron JM, Ibrahim SS, Irving H, Barnes KG, Mukwaya LG, et al. Widespread pyrethroid and DDT resistance in the major malaria vector Anopheles funestus in East Africa is driven by metabolic resistance mechanisms. PloS one. 2014;9(10):e110058.

89. Ochomo E, Bayoh NM, Kamau L, Atieli F, Vulule J, Ouma C, et al. Pyrethroid susceptibility of malaria vectors in four Districts of western Kenya. Parasites & Vectors. 2014;7(1):310.

90. Tangena J-A. The risk of vector-borne disease exposure in rubber plantations of northern Lao PDR: Durham University; 2016.

91. Pichler V, Bellini R, Veronesi R, Arnoldi D, Rizzoli A, Lia RP, et al. First evidence of resistance to pyrethroid insecticides in Italian Aedes albopictus populations 26 years after invasion. Pest Manag Sci. 2018;74(6):1319-27.

92. Waits CM, Fulcher A, Louton JE, Richardson AG, Becnel JJ, Xue R-d, et al. A comparative analysis of resistance testing methods in Aedes albopictus (Diptera: Culicidae) from St. Johns County, Florida. Florida Entomologist. 2017;100(3):571-7.
